# Pseudonormal Morphology of Salivary Gland Adenoid Cystic Carcinoma Cells Subverts the Antitumor Reactivity of Immune Cells: A Tumour‐Cell–Based Initiation of Immune Evasion

**DOI:** 10.1002/cnr2.70019

**Published:** 2024-09-26

**Authors:** Rajdeep Chakraborty, Thiri Zaw, Pallavi Khodlan, Charbel Darido, Giuseppe Palmisano, Arthur Chien, Aidan Tay, Shoba Ranganathan, Fei Liu

**Affiliations:** ^1^ Applied Biosciences, Faculty of Science and Engineering Macquarie University Sydney New South Wales Australia; ^2^ School of Natural Sciences, Faculty of Science and Engineering Macquarie University Sydney New South Wales Australia; ^3^ Australian Proteome Analysis Facility, Faculty of Science and Engineering Macquarie University Sydney New South Wales Australia; ^4^ Peter MacCallum Cancer Centre Melbourne Victoria Australia; ^5^ Sir Peter MacCallum Department of Oncology The University of Melbourne Melbourne Victoria Australia; ^6^ GlycoProteomics Laboratory, Department of Parasitology ICB, University of Sao Paulo São Paulo São Paulo Brazil; ^7^ Australian e‐Health Research Centre, Transformational Bioinformatics Group CSIRO New South Wales Australia

**Keywords:** adenoid cystic carcinoma, anti‐tumour reactivity, apoptosis, cancer, Gipie, head and neck cancer, immunotherapy, mucoepidermoid carcinoma, oral cancer, oral squamous cell carcinoma

## Abstract

**Introduction:**

Salivary gland adenoid cystic carcinoma (ACC), mucoepidermoid carcinoma (MEC) and oral squamous cell carcinoma (OSCC) occurs within the head and neck region. So far immune check point inhibitors failed in ACC. Gipie (CCDC88B) is a microtubule linker protein that activates immune cells. Gipie expressions found in head and neck cancer cells. We hypothesised that the presence of Gipie diminishes anti‐tumour reactivity of immune cells towards head and neck cancer.

**Method:**

To determine the effect of Gipie in oral and salivary gland cancer cells, Gipie was silenced in cancer cells in cancer‐immune cells co‐culture models and we performed 3D Z series confocal imaging, annexin V and immune activation flow cytometry, proteome profiler and discovery phase proteomics.

**Results:**

ACC cells morphed into pseudonormal morphology in immune co‐culture models. Silencing Gipie in ACC cells showed significant increase of apoptotic cells and activated natural killer cells, and lowering of regulatory T cells. Other salivary and oral cancer cells showed negligible effect of Gipie. Proteome profiler and proteomics assay confirmed Gipie affecting proliferation mechanism and immune activated proteins in ACC immune co‐culture models.

**Conclusion:**

Overall, we conclude that the presence of Gipie has a confounding role during the ACC–immune cell interaction.

**Summary**

- Adenoid cystic carcinoma cells morphed to pseudonormal morphology after interaction with immune cells.
- Silencing Gipie in ACC increased antitumour reactivity of immune cells leading to increased apoptosis of ACC.
- Phosphokinase array investigation elucidated the Gipie proliferation and apoptotic interacting partners in ACC.
- Upregulation of granzymes and CD48 in activated immune cells in Gipie‐silenced ACC immune co‐culture model.

## 1 Introduction

Adenoid cystic carcinoma (ACC) is the second most common salivary gland cancer affecting the head and neck region, after mucoepidermoid carcinoma [1]. The ACC tumour microenvironment shows low levels of CD8+ tumour infiltrating lymphocytes, PD‐1 and cytotoxic T lymphocytes antigen 4 (CTLA‐4) positive cells and overexpresses immune inhibitors such as PD‐L2, but not PD‐L1 [2]. Signalling molecules of the Wnt/β‐catenin pathway and associated proteins like galectin‐3 and cyclin D1 are responsible for the aggressive phenotype of ACC [3].

Oral cancer, or oral squamous cell carcinoma (OSCC), is the most common head and neck cancer, with the most commonly affected region being the tongue [4]. Proteins including EGF, STAT3, the ErbB proteins, GRB2, Shc, SOS1, Ras, Raf, MAPK, MEK/ERK, PI3K/Akt and cyclin D1 are responsible for the progression of OSCC [4].

Immune cells, including macrophages, neutrophils, dendritic cells, cytotoxic T cells and natural killer (NK) cells are involved in restricting cancer progression [5]. Highly immunogenic cancer cells evade immune‐mediated killing by disabling components of the immune machinery [5].

Pembrolizumab has been beneficial in recurrent, unresectable and metastatic OSCC. The success of ongoing immunotherapy (pembrolizumab, PD‐1 inhibitor) in OSCC has placed PD‐1/PD‐L1 in the spotlight for OSCC treatment, making it a prospective biomarker of OSCC in future [6].

However, immunotherapy is largely ineffective in ACC. Clinical trials of pembrolizumab in ACC have shown no appreciable tumour response [7]. Endeavours to harness the immune system to restrict ACC progression continue, with laboratory‐based research exploring the molecular mechanisms of ACC cell–immune cell interactions [7].

The Hook‐related protein family (HkRPs), like the Hook family, are proteins that link cytoplasmic organelles to microtubules. Gipie (GRP78‐interacting protein induced by ER stress, also known as HkRP3, CCDC88B and FLJ00354) is one of the HkRPs [8]. Gipie is also associated with T cell maturation and inflammatory function [9], and regulation of microtubule‐mediated lytic granule clustering and subsequent target cell killing in NK cells [10]. Additionally, Gipie mutations are strongly associated with inflammatory bowel disease and leprosy [11].

The molecular function of Gipie is still largely elusive; however, surprisingly, Gipie is expressed in oral cancer and salivary gland cancer cells [12]. This led us to hypothesise that Gipie helps ACC to survive and proliferate by avoiding immune attack. In this study, we explored the effect of Gipie on the protein expressions in immune cells in a 3D oral cancer/salivary gland cancer—immune co‐culture model.

## 2 Methods

### 2.1 3D Oral and Salivary Gland Cancer‐Immune Cell Co‐Culture

3D oral and salivary gland cancer‐immune co‐culture models were made using UM‐HACC‐2A (adenoid cystic carcinoma) (Cat T8326) (abm) cells, A‐253 (mucoepidermoid carcinoma) (HTB‐41, ATCC) cells, CAL27 (CRL‐2095, ATCC), SCC4 (CRL‐1624, ATCC), SCC9 (CRL‐1629, ATCC), SCC25 [CRL‐1628, ATCC] (OSCC) cells and OKF6 [CVCL_L225] (normal oral) cells. The cells were co‐cultured in presence of NK‐92 (natural killer) [CRL‐2407, ATCC] cells and Jurkat, Clone E6‐1 (acute T cell leukaemia) [TIB‐152, ATCC] cells. Cell culture inserts (transparent PET membrane) 0.4 μm 20 × 25 mm (Thermo Fisher Scientific, Cat NUN140640), Falcon cell culture insert (transparent PET membrane) six‐well 3.0 μm (In Vitro Technologies, Cat 353091) and Falcon cell culture insert (transparent PET membrane) six‐well 0.4 μm (In Vitro Technologies, Cat 353090) were used for co‐culturing. Details of the cell lines and medium used are provided in Table S1.

### 2.2 Expression Validation and Silencing of Gipie

Western blot was done using Anti‐*CCDC88B* antibody produced in rabbit (Sigma Aldrich, Cat HPA026652‐25 μL) and LC/MS/MS done to determine the protein expression of Gipie in cell lines. Silencing of Gipie (CCDC88B) was done by 1 nmol of *CCDC88B* Silencer pre‐designed siRNA (Ambion, Cat AM16704). Quality of the experiments were maintained by using negative and positive silencing controls.

### 2.3 Morphology and Transmigration in a Co‐Culture Setup

The cell insert membranes were retrieved from the 3D‐co‐culture models and they were fixed by 4% paraformaldehyde for 30 min. Followed by incubation with Actigreen 488 (Invitrogen, Cat R37110) for 30 min and then DAPI counterstained with fixative with ProLong Glass antifade mountant with NucBlue (Invitrogen, Cat P36983). The morphology and transmigration 3D Z series convoluted images and videos were acquired using FLUOVIEW FV3000 (Olympus, Japan).

### 2.4 Proliferation and Apoptosis Assay

The cancer cells were detached using Accutase (Sigma Aldrich, Cat A6964). Annexin V flow cytometry was done using TACS+ Annexin FITC (R&D Systems, Cat 4830‐01‐1) and 10× propidium iodide (R&D Systems, Cat 4830‐01‐3) according to the manufacturer instruction. Ten thousand events flow cytometry data of the prepared samples were acquired from BD Accuri C6 Plus Flow Cytometer (BD Biosciences). Flow cytometry analysis was done in FlowJo 10.8.1 (Becton Dickinson & Company, USA).

### 2.5 NK Activation and Regulatory T Cell Assay

NK and JK cells collected from the inserts after predetermined incubation time. Anti‐hNKp30/NCR3 PE‐conjugated (R&D Systems, Cat FAB1849P) and anti‐hIFNγ‐Alexa Fluor 488‐conjugated antibody (R&D Systems, Cat IC285G) (5 μL/sample) used for NK activation flow cytometry assay. R&D Systems FlowX Human Regulatory T Cell MultiColor Flow Kit (R&D Systems, Cat RDSFMC021) was used to determine the percentage of regulatory T cells.

### 2.6 Proteome Profiler

Proteome Profiler Human Phospho‐Kinase Array Kit (R&D Systems, Cat RDSARY003C) was used to identify the Gipie‐affected phosphorylated proteins. Proteome Profiler Human Apoptosis Array Kit (R&D Systems, Cat RDSARY009) was used to identify the Gipie‐affected phosphorylated pro apoptotic and anti‐apoptotic proteins. All materials used during the project are provided in the Table S2.

### 2.7 SWATH LC–MS/MS Analysis

SWATH analysis of the acquired cell samples from 3D‐cancer immune co‐culture models were done using Triple TOF 6600 mass spectrometer. SWATH data processing were done using PeakView 2.2. The multiple comparison of different samples was done using GenePattern server (SwathPairsAndOverall) (Australian Proteome Analysis Facility, Macquarie University).

### 2.8 Bioinformatics Analysis

Protein interaction network was visualised and analysed using STRING: functional protein annotation networks https://string‐db.org/, Cytoscape 3.9.1 www.cytoscape.org and the database for annotation, visualisation and integrated discovery (DAVID) https://david.ncifcrf.gov/.

### 2.9 Statistical Analysis

Statistical analysis was performed using Graph Pad Prism version 9 (GraphPad software, San Diego, CA, USA). The data were tested for normality of distribution using D'Agostino and Pearson omnibus normality test. One‐way ANOVA (analysis of variance) Kruskal–Wallis test multiple comparison on mean ranks were performed in the salivary gland and oral cancer apoptosis test, survival assay, NK cell and Jurkat cell activation assays. Paired *T*‐test Wilcoxon matched‐pairs signed rank test were performed in multiple reaction monitoring targeted phase proteomic investigation of immune activated proteins. The probability threshold of *p* ≤ 0.05 was considered statistically significant.

## 3 Results

### 3.1 Gipie Expression in Immune Cells and Cancer Cells

The project began with an investigation of Gipie (CCDC88B) expression; mass spectrometry‐based proteomics (SWATH LC MS/MS) and western blots were used to determine the silencing of Gipie in immune cells and cancer cells (Figure S1). We already found Gipie protein expression in immune cells, oral cancer and salivary gland cancer cells (adenoid cystic carcinoma and mucoepidermoid carcinoma) [12]. Additionally, mass spectrometry confirmed protein expressions of Gipie in cell lines used during the project. However, negligible Gipie protein levels were found in OKF6 cells.

### 3.2 Cancer–Immune Cell Interactions Affect the Morphology of ACC Cells

ACC cells are fibroblast‐like in form (Figure S2). ACC–immune cell interaction led to an interesting observation, in that the ACC cells altered to show a pseudo‐normal morphology (undefined spindle/slender) after interacting with both NK and JK cells in the 3D co‐culture model (Figure S2). Gipie‐silenced ACC cells showed a lymphoblast like (spherical) morphology (Figure S2). Negligible changes were seen in the morphology of MEC cells and all the oral cancer cells in a similar experimental set‐up (Figure S2). This suggests that ACC cells behave differently in the presence of immune cells compared to other head and neck cancer cells.

### 3.3 Modification of Gipie Expression Affects the Percentage of Apoptotic Cells

In NK co‐culture models, a significant increase (five‐fold) in apoptotic cells was seen in Gipie‐silenced UM HACC‐2A cells compared to their unaltered counterparts (Figure S3). Apart from ACC cells, no other cell type showed any significant change in apoptosis profile after Gipie silencing compared to their unaltered counterparts (Figure S3). The apoptotic profiles of all the cancer and normal cells tested in the JK co‐culture model showed similar results to those seen in the NK co‐culture model (Figure S4).

### 3.4 Gipie Affected Phosphorylated Proteins

**FIGURE 1:**
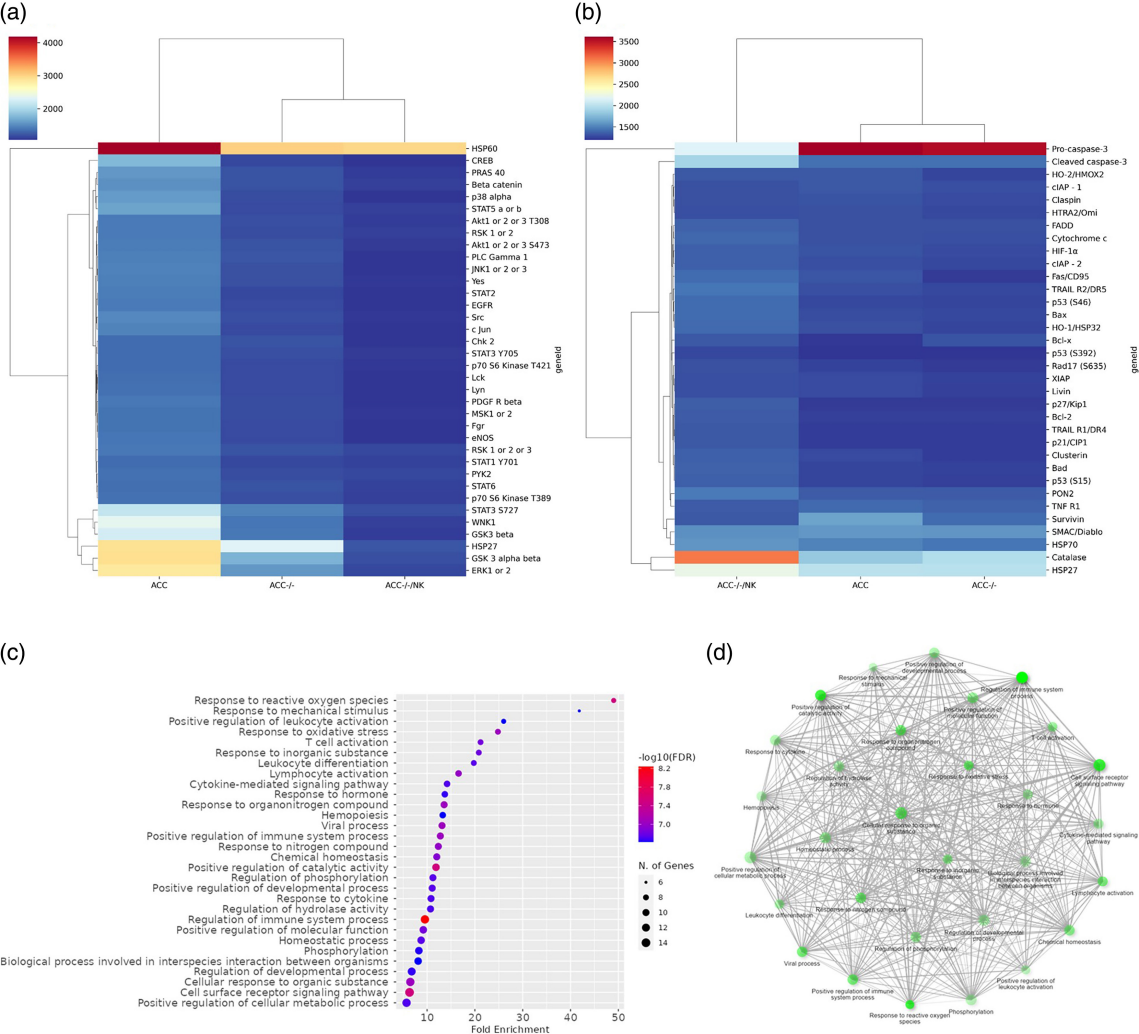
Gipie expression modification affected the proliferation and apoptosis related proteins in a co‐culture model. (a) and (b) heat map to visualise hierarchical clustering of upregulated and downregulated proliferation and apoptotic pathways related protein affected by Gipie in ACC, respectively. (c) Dot plot analysis of overrepresented Gipie‐affected proliferation and apoptosis related protein pathways in UM‐HACC‐2A. (d) Interactive static plot shows the relationship between enriched pathways.

**FIGURE 2:**
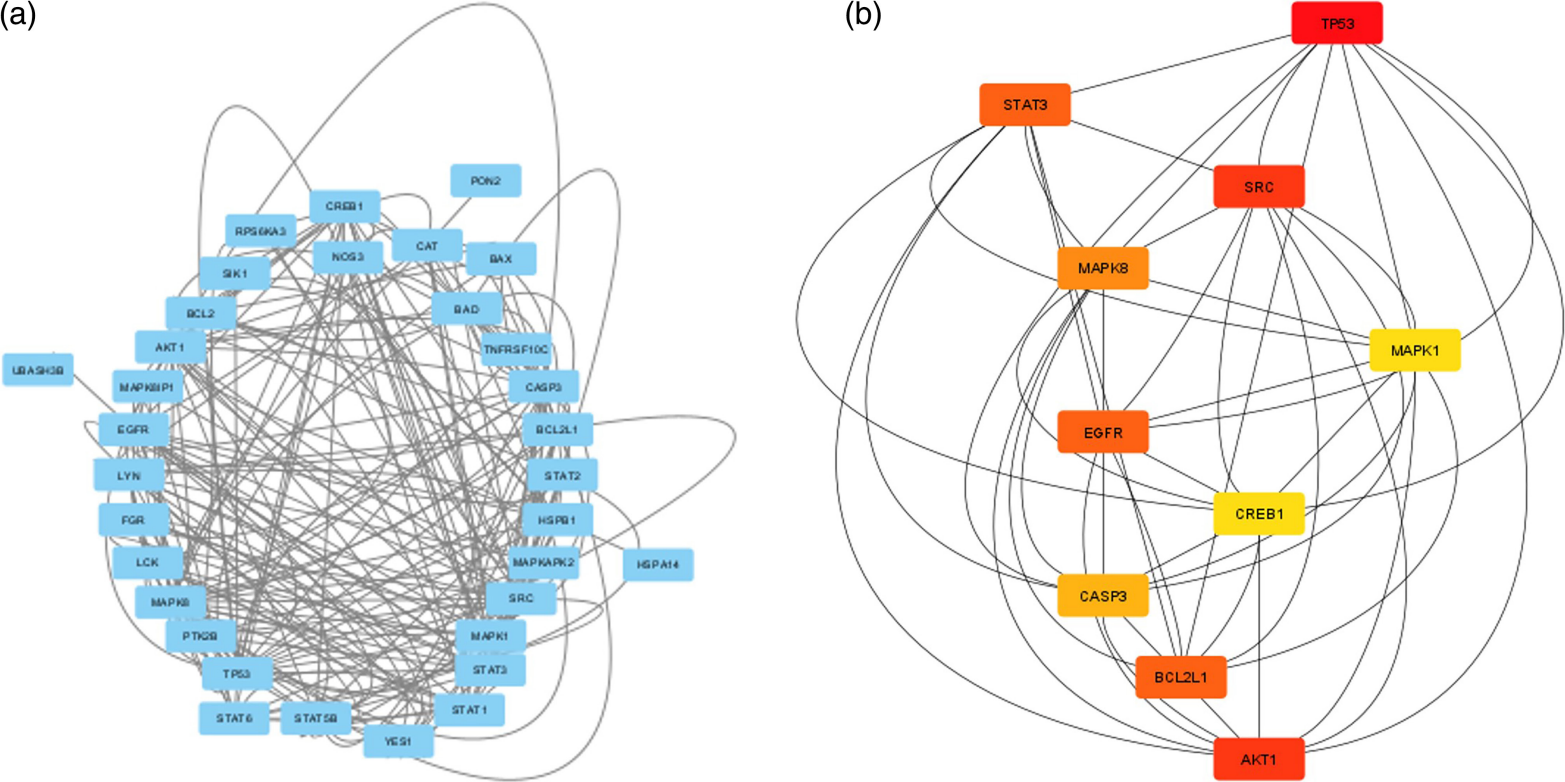
Gipie expression modification affected the phosphorylated protein interaction in a co‐culture model. (a) STRING protein–protein interaction network generated using Cytoscape 3.9.1 www.cytoscape.org. The circular interaction network of phosphorylated proteins significantly affected by Gipie in adenoid cystic carcinoma. (b) Visualisation of the top 10 ranks (closest interacting and significantly affected proliferation and apoptosis related proteins affected by Gipie in ACC) achieved after ranking shortest path based on degree using *cytoHubba* control panel in Cytoscape 3.9.1 www.cytoscape.org.

Gipie affected 48 phosphorylated proteins (Figure 1a,b). After screening based on the degree of closeness, 33 proteins were selected from the STRING domain (Figure S4). Among the closest interacting phosphorylated proteins, CREB1, CAT, EGFR, STAT2, STAT3, LYN, AKT1, STAT6, STAT1, LCK, FGR, SRC, MAPK and YES1 are proliferation‐related proteins, whereas CASP3, BCL2, BAD, BAX and TP53 are pro‐apoptotic proteins, and PON2 is anti‐apoptotic (Figure 2a). The top 10 rank‐based results signifying that proliferation‐related, pro‐apoptotic and anti‐apoptotic proteins interact and affect cancer–immune cell interactions (Figure 2b).

Additional proliferation‐related phosphorylated proteins that did not interact closely with other affected proteins, but that were significantly affected by Gipie in ACC are p38α, JNK, PDGF Rβ, WNK1, PLC γ1, GSK3β, eNOS, PYK2, Chk2, HSP60, RSK, PRAS40, β catenin and TRAIL R1. The Gipie‐silenced ACC cells from immune co‐cultures showed a significant reduction in phosphorylated proliferation‐related proteins (Figure 1a). This observation is concordant with our previous finding of lower survival rates in Gipie‐silenced ACC cells cultured in the presence of immune cells.

Interestingly, the pro‐apoptotic factor, procaspase 3, was significantly reduced, but at the same time, cleaved caspase 3 was significantly higher in Gipie‐silenced ACC cells in immune co‐culture (Figure 1b). This is most likely due to the conversion of procaspase to cleaved caspase during activation. This finding supports our observation of high levels of apoptosis in the co‐culture experiments.

We next carried out protein interaction, gene enrichment and functional mapping analysis, and found that most of the affected proteins participate in the survival, proliferation and apoptosis pathways of ACC cells (Figures S5–S9). Unexpectedly, a large set of these proteins also affect immune system processes and cell surface receptor signalling pathways (Figure 1c,d).

### 3.5 Regulatory T Cell and NK Cell Activation

A four‐fold higher FOXP3/IL2Rα/CD25 positive JK cells was seen in the control co‐cultures compared to the Gipie‐silenced co‐cultures (Figure S10). That indicates a reduction in immune suppressive cells after Gipie silencing in UM‐HACC‐2A cells. JK cells from UM‐HACC‐2A and A‐253 co‐culture models showed significant higher levels of immunosuppressive cells compared with that seen in OKF6 co‐cultures (Figure S10), further suggesting that salivary gland cancer cell lines and OSCC cell lines behave differently towards immune cells.

NKp30/IFNγ positive NK‐92 cells were four‐fold higher in Gipie‐silenced UM‐HACC‐2A co‐culture models compared with NK‐92 cells in the comparator unaltered co‐culture model (Figure S10). This is consistent to our previous findings showing Gipie silencing downregulates immune suppression and survival and upregulates apoptosis [12].

Gipie‐silenced UM‐HACC‐2A, A‐253, SCC9 and CAL27 showed significantly higher numbers of NKp30/IFNγ positive NK‐92 cells compared with OKF6 co‐cultures (Figure S10).

In all cancer cell line–JK and NK immune co‐culture models we found that the unaltered UM‐HACC‐2A and Gipie‐silenced UM‐HACC‐2A cluster is well delineated compared with other cancer cell lines in different co‐culture conditions (Figures S10 and S11). This distance between the immune cell expressions of Gipie‐silenced UM‐HACC‐2A, UM‐HACC‐2A and both OKF6 conditions, and all other cancer cell lines suggests that Gipie functions best in the UM‐HACC‐2A cells in terms of immune cell anti‐tumour reactivity.

### 3.6 Immune Cell Activated Proteins

In each experimental run, a total of four experimental groups, comprising three technical replicates of each group: namely, (a) JK cells from the unaltered UM‐HACC‐2A co‐culture model, (b) JK cells from the Gipie‐silenced UM‐HACC‐2A model, (c) NK‐92 cells from the unaltered UM‐HACC‐2A co‐culture model and (d) NK‐92 cells from the Gipie‐silenced UM‐HACC‐2A model.

**FIGURE 3:**
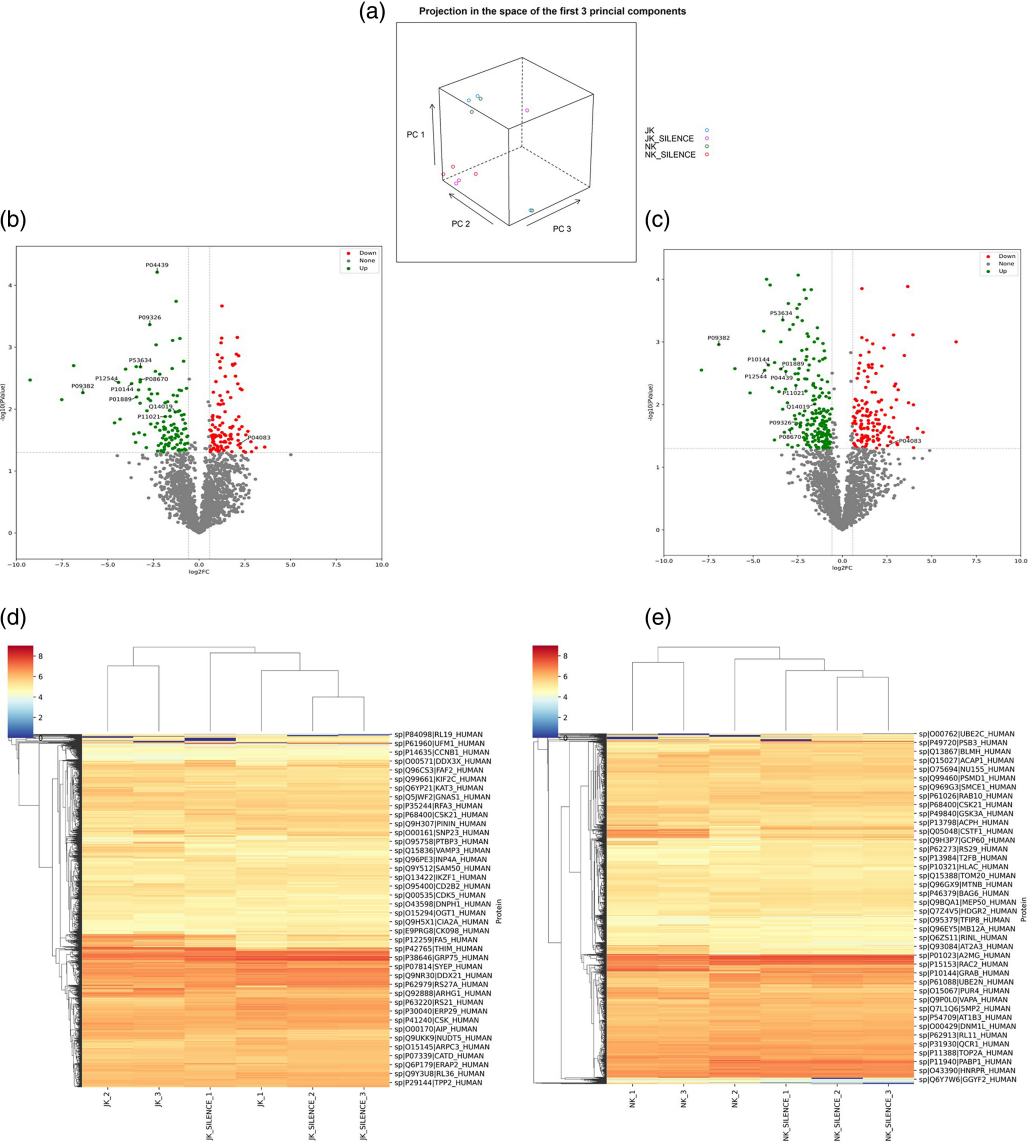
Differential expressions of immune activated protein in activated immune cells due to silencing of Gipie in ACC co‐culture model. (a) PCA 3D plots of upregulated and downregulated proteins in JK and NK cells due to the effect of Gipie in UM‐HACC‐2A cells. JK from UM‐HACC‐2A co‐culture (blue), JK from Gipie‐silenced UM‐HACC‐2A co‐culture (pink), NK from UM‐HACC‐2A co‐culture (green), NK from Gipie‐silenced UM‐HACC‐2A co‐culture (red). (b) JK cells from Gipie‐silenced UM‐HACC‐2A co‐culture group. (c) NK cells from Gipie‐silenced UM‐HACC‐2A co‐culture group. Heat map to visualise hierarchical clustering of upregulated and downregulated immune proteins in, (d) JK, (e) NK, in Gipie‐silenced UM‐HACC‐2A—immune co‐culture model.

On principal component analysis of the mass spectrometry results, we found clear separation of the Gipie‐silenced and unaltered cohorts. The NK‐92 cells from the experimental groups showed consistent data points, while several of the JK isolates from the Gipie‐silenced group were not as consistent (Figure 3a).

A total 2176 proteins were identified during the discovery phase mass spectrometry‐based proteomic analysis. With UM‐HACC‐2A cells, Gipie‐induced downregulation of 601 and 561 JK and NK‐92 proteins, respectively, and upregulation of 634 and 681 JK and NK‐92 proteins. In both JK and NK‐92 cells, most of the upregulated proteins were related to the immune response, while interestingly, most of the downregulated proteins positively influence the anti‐tumour reactivity of immune cells (Figures S12–S14).

Next, all data were analysed using multiple comparison ANOVA *p*‐values adjusted for multiple testing by Benjamin–Hochberg FDR adjustment. The significant differentially expressed proteins based on fold change and *p*‐value cutoffs were considered for further analysis (Figure S15).

We identified 336 differentially expressed proteins common to both JK and NK‐92 cells (Figure 3b,c). The overexpressed proteins are all associated with the immune response, lymphocyte‐mediated cytotoxicity, leukocyte homeostasis, leukocyte activation, immune activation, lymphocyte co‐stimulation, antigen processing and presentation (Figure 3d,e).

**FIGURE 4:**
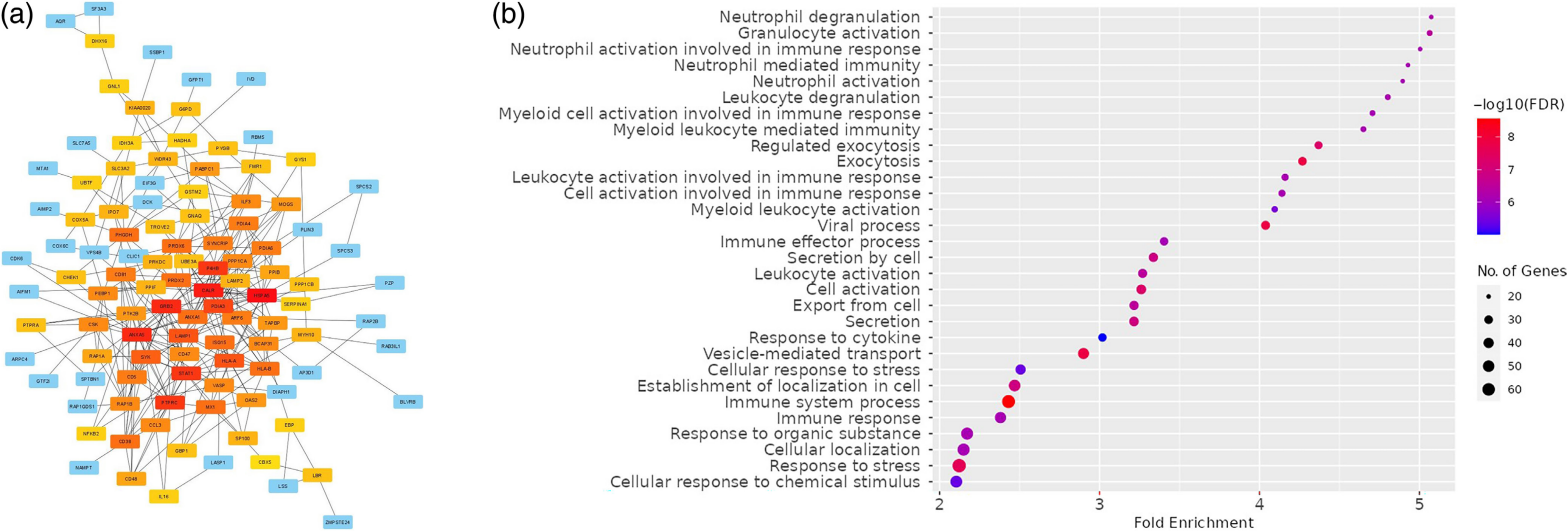
Protein interaction and GO enrichment pathway of immune activated protein in activated immune cells due to silencing of Gipie in ACC. (a) Protein interaction of significantly altered protein in activated immune cells. (b) GO enrichment analysis of Gipie‐affected immune proteins related protein pathways.

The closest protein network analysis implies that the Gipie‐affected immune‐activated proteins, which are most likely responsible for the increase of anti‐tumour activity of immune cells against UM‐HACC‐2A, interact closely with each other (Figures 4a, S16 and S17). Subsequently, we performed a gene enrichment analysis that further highlighted the dominance of the immune activation process in immune cells from Gipie‐silenced co‐cultures (Figures 4b and S18–S20).

## 4 Discussion

The presence of immune proteins in cancer cells affects cellular morphology. Previously, it has been found that platelet‐derived MHC class I conferred a pseudo‐normal morphology on cancer cells, and that this subverted the anti‐tumour reactivity of immune cells [13].

Microtubule‐associated proteins may be fundamental regulators of cancer cell aggressiveness, given their role in the alteration of microtubule dynamics, cancer migration, invasion and the epithelial‐mesenchymal transition [14]. Interestingly, when Gipie was silenced in UM‐HACC‐2A cells, no significant changes were observed in the rate of apoptosis. However, when the same experiment was performed in a co‐culture set‐up; that is, in presence of immune cells, a significant increase in the numbers of apoptotic cells was observed. Indeed, either the paracrine effect or chemoattractant released by the cancer cells as a result of downregulation in Gipie expression attracts immune cells [15, 16, 17]. These chemoattractants have previously been shown to be modified by microtubule linker proteins. Further, actin is intimately involved in the regulation and trafficking of chemoattractants like CXCR1 and CXCR2 [18]. Taken together, this suggests a possible role of Gipie in modifying actin and thus impacting the release of chemoattractants, which then inhibit immune cell reactivity against UM‐HACC‐2A cells. This effect is reversed after silencing Gipie in cancer cells.

An increase in the survival of cancer cells was observed after silencing Gipie in immune cells. That is possibly due to immune cells dysfunction due to the absence of Gipie, which is required by immune cells for activation, proliferation, migration and survival. Avoiding immune‐mediated attack is one of the hallmarks of cancer, and also of ACC. Similar to Gipie, other microtubule proteins, such as tubulin, also play an active role in the innate immune system [19].

STAT3 inhibition results in reduction in proliferation and invasion of ACC [20]. Additionally, HSP60 and HSP27 are negatively correlated with prognosis in ACC. Previously both these heat shock proteins have been seen to aid ACC proliferation, invasion and to confer resistance to radiotherapy [21]. Further, ERK overexpression in ACC increases disease progression and metastasis [22]. However, the function of CREB and WNK in ACC is still yet to be elucidated.

Surprisingly, along with the pro‐apoptotic proteins, anti‐apoptotic proteins were also affected by the absence of Gipie. Upregulation of PON2, clusterin, Rad 17, p27 and p21 was observed in Gipie‐silenced ACC cells in immune co‐cultures. However, survivin, which is highly expressed in ACC, was underexpressed in the Gipie‐silenced cohort. This supports the reduction in survival in the Gipie‐silenced cohort [23].

Based on the possible role of Gipie in the cancer–immune cell axis, we conducted immune activation assays. Gipie‐silenced cultures showed a significant reduction in FOXP3/IL‐2Rα/CD25 positive cells. JK E6.1 cells have previously been shown to lack expression of IL‐2Rα/CD25 [24]. However, these cells expressed this receptor when they were stimulated with T cell receptor early signalling complex proteins [25]. Early signalling complex proteins include LCK (one of the Gipie‐affected proteins), which ultimately activates the ERK/MAPK pathway. It is interesting to note that early signalling complex proteins also contribute to the signalling pathways of chemoattractants, such as CXCR4. This strongly indicates the presence of a microtubule linker protein, actin cytoskeleton, early signalling complex protein and chemoattractant axis. Additionally, cellular stress has previously been demonstrated to induce the expression of IL‐2Rα/CD25 in JK E6.1 cells [26].

NK cells demonstrated significantly higher Nkp30/IFNγ expression in Gipie‐silenced ACC–immune co‐cultures. This suggests an increase of activated immune cells in the Gipie‐silenced cohort. Upregulation of IFNγ was later confirmed by a significant increase in STAT1 protein expression in NK cells from the Gipie‐silenced cohort compared to NK cells from the control cultures. STAT1 is one of a potential marker and binding partner of IFNγ.

IFNγ also upregulates pro‐apoptotic factors in cancer cells [27]. Again, this is in accordance with our results, where hyperactivation of NK‐92 cells and upregulation of pro‐apoptotic factors was seen in Gipie‐silence co‐cultures. Furthermore, changes in the actin cytoskeleton may upregulate IFNγ production in the immune cells [28].

IFNγ has been shown to induce immunosuppression in ACC [29]. However, our results conflict with these findings. Silencing Gipie resulted in higher expression levels of immune proteins such as interleukins, complement activation proteins and granzymes, among others. Granzyme A and Granzyme B showed significant upregulation in immune cells. That is also possibly due to the significant upregulation of dipeptidyl peptidase 1. Previously, dipeptidyl peptidase 1 has been shown to be involved in the synthesis of granzymes A and B [30]. This further confirms the cytotoxic effect of immune cells towards Gipie‐silenced ACC cells. The upregulation of HLA antigens indicates an active process of recognition in the immune cells, although it is difficult to conclude how these HLA antigens function in the ACC–immune cell axis. Galectin 1 from fibroblasts and TGFβ have previously been shown to synergistically foster cancer cell proliferation and metastasis [31]. Our results contrast with this finding, although we are not able to confirm the role of TGFβ in ACC. Vimentin has been previously seen to function in the activation of immune cells, but it also induces apoptosis in these cells [32]. At this preliminary stage, we cannot confirm the role of vimentin in ACC–immune cell interactions. Finally, the significant upregulation of endoplasmic reticulum chaperone BiP further confirms the consistent upregulation of Gipie in activated cells. BiP and GRP78 are binding partners of Gipie.

## 5 Conclusion

Despite getting the preliminary indications, we could neither determine the exact immune proteins that affects cellular morphology of ACC, nor we could determine the specific factors from ACC that induce Treg‐like features of JK cells. In future, to determine the paracrine effect of immune cells on cancer cells and vice versa, supernatant from each cell will be collected and discovery phase mass spectrometry will be performed.

Overall, we conclude that Gipie indeed affect proliferation mechanism and plays a confounding role during cancer–immune cell interaction. Although we are still not sure regarding the exact mechanism of Gipie affecting the immune evasion. This project is successful in showing the cancer‐immune cell interaction in ACC, which opens new possibilities for future research and development of immunotherapy for ACC. This project acts as a prelude to address the critical question of immunotherapy failure in adenoid cystic carcinoma. Determining immune activation protein changes due to the presence and absence of Gipie in ACC cells will eventually lead to the elucidation of the role of hook‐related proteins in inhibition of anti‐tumour reactivity of immune cells that will eventually help dissecting the underlying mechanisms contributing to immunotherapy treatment resistance. Thus, it will provide new mechanistic insight for improving the efficacy of immunotherapies in ACC. Additionally, this project will help identify novel precision‐based treatment strategy to restrict the progression of ACC, capitalising on clinically relevant 3D cancer—immune co‐culture model that closely resembles an in vivo state without the need for animal models.

## Author Contributions

**Rajdeep Chakraborty:** conceptualization, methodology, software, data curation, formal analysis, project administration, resources, visualization, validation, investigation, funding acquisition, writing – original draft. **Thiri Zaw:** data curation, methodology, investigation, validation, formal analysis, supervision, resources, visualization, writing – review and editing. **Pallavi Khodlan:** software, data curation, formal analysis, investigation, visualization, writing – review and editing. **Charbel Darido:** methodology, validation, formal analysis, supervision, resources, writing – review and editing, visualization. **Giuseppe Palmisano:** methodology, software, investigation, validation, formal analysis, supervision, resources, visualization, writing – review and editing. **Arthur Chien:** methodology, software, data curation, investigation, validation, formal analysis, visualization, resources, writing – review and editing. **Aidan Tay:** software, investigation, validation, formal analysis, visualization, resources, writing – review and editing. **Shoba Ranganathan:** methodology, investigation, validation, supervision, visualization, resources, writing – review and editing. **Fei Liu:** methodology, software, investigation, validation, formal analysis, supervision, resources, visualization, writing – review and editing.

## Disclosure

Australian and New Zealand Head and Neck Cancer Society is a non‐profit philanthropic society. The society's goal is to achieve greater prevention and early detection of head and neck cancer. The society supported the project to encourage adenoid cystic carcinoma research in Australia.

## Ethics Statement

The entire work was done following the biosafety approval by the Macquarie University Biosafety committee (Mammalian Cell Culture 5215).

## Conflicts of Interest

The authors declare no conflicts of interest.

## Supporting information


**Data S1.** Supporting information.

## Data Availability

The data that support the findings of this study are openly available in figshare repository at https://doi.org/10.6084/m9.figshare.24546859.
