# Supplementary material for: Pseudonormal Morphology of Salivary Gland Adenoid Cystic Carcinoma Cells Subverts the Antitumor Reactivity of Immune Cells: A Tumour‐Cell–Based Initiation of Immune Evasion
Source: Cancer Rep (Hoboken). 2024 Sep 26;7(9):e70019. doi: 10.1002/cnr2.70019 (PMC11425664; doi:10.1002/cnr2.70019)
Supplement: Supplementary file 1 — Data S1. Supporting information. [file CNR2-7-e70019-s001.docx]

**SUPPLEMENTAL FILE**

**Pseudonormal morphology of salivary gland adenoid cystic carcinoma cells subverts the antitumor reactivity of immune cells: A tumour-cell based initiation of immune evasion**

Rajdeep Chakraborty^1,2*^, Thiri Zaw^3^, Pallavi Khodlan^1^, Charbel Darido^4,5^, Giuseppe Palmisano^2,6^, Arthur Chien^2^, Aidan Tay^1,7^, Shoba Ranganathan^1^, Fei Liu^2^

^1^Applied Biosciences, Faculty of Science and Engineering, Macquarie University, Sydney, NSW 2109, Australia

^2^School of Natural Sciences, Faculty of Science and Engineering, Macquarie University, Sydney, NSW 2109, Australia

^3^Australian Proteome Analysis Facility, Faculty of Science and Engineering, Macquarie University, Sydney, NSW 2109, Australia

^4^Peter MacCallum Cancer Centre, Melbourne, VIC 3000, Australia

^5^Sir Peter MacCallum Department of Oncology, The University of Melbourne, Melbourne, VIC 3000, Australia

^6^GlycoProteomics Laboratory, Department of Parasitology, ICB, University of Sao Paulo, São Paulo 05508-000, SP, Brazil

^7^Australian e-Health Research Centre, Transformational Bioinformatics Group, CSIRO, New South Wales, Australia

*Correspondence to: [rajdeep.chakraborty@hdr.mq.edu.au](mailto:rajdeep.chakraborty@hdr.mq.edu.au)

**Supplementary Table 1. Oral Cancer and salivary gland cell lines**

|  | **OKF6** | **SCC4** | **SCC9** | **SCC25** | **CAL 27** | **UM-HACC-2A** | **A-253** |
| --- | --- | --- | --- | --- | --- | --- | --- |
| **Source** | Cellosaurus | ATCC | ATCC | ATCC | ATCC | The University of Michigan;  abm^®^ Canada | ATCC |
| **Specimen site** | Floor of mouth ; normal oral keratinocytes | Tongue SCC | Tongue SCC | Tongue SCC | Tongue SCC | Minor salivary gland  at the base  of the tongue | submaxillary  salivary gland |
| **Type** | Immortalised  normal oral cells | Immortalised  oral cancer cells | Immortalised  oral cancer cells | Immortalised  oral cancer cells | Immortalised  oral cancer cells | Primary | Immortalised  mucoepidermoid carcinoma cells |
| **Treatment** | No | Radiation  & methotrexate | No | No | No | No | No |
| **Sex** | Male | Male | Male | Male | Male | Female | Male |
| **Age (years)** | 57 | 55 | 25 | 70 | 56 | 53 | 54 |
| **TNM staging** | - | T3N0M0 | T2N1 | T1N1M0 | - | T3N1M0 | - |
| **Culture media** | K-SFM media  + growth factors | DMEM  + 10% FBS | DMEM  + 10% FBS | K-SFM media  + growth factors | DMEM  + 10% FBS | Optimised  salivary gland  medium | McCoy’s 5A  medium  +10% FBS |
| **Freezing media** | 50% FBS  + 10% DMSO + DMEM | 50% FBS  + 10% DMSO  + DMEM | 50% FBS +  10% DMSO + DMEM | 20% FCS +  20% DMSO + DMEM/F12 | 50% FBS +  10% DMSO + DMEM | Optimised  Cryopreservative  Medium | Optimised  Cryopreservative  Medium |

**Supplementary Table 2. All reagents used during the project**

| **Cat No.** | **Name of the reagent** |
| --- | --- |
| TM003 | PriGrow III |
| G255 | Penicillin/Streptomycin Solution |
| TM024 | Cryopreservation Medium |
| 4569033 | Any kD Mini-PROTEAN TGX Precast Protein Gels, 10-well, 30 µl Pkg of 10, Any kD p recast polyacrylamide gel, 8.6 × 6.7 cm (W × L), for use with Mini-PROTEAN Electrophoresis Cells |
| 1610747 | 4x Laemmli Sample Buffer 10 ml, premixed 4x Laemmli protein sample buffer for SDSPAGE |
| 1610732 | 10x Tris/Glycine/SDS Pkg of 1, 1 L, 10x premixed electrophoresis buffer, contains 25 m M Tris, 192 mM glycine, 0.1% SDS, pH 8.3 following dilution to 1x with water |
| 1705060 | Clarity™ Western ECL Substrate, 200 ml |
| 1703966 | Extra Thick Blot Filter Paper, Precut, 7 x 8.4 cm Pkg of 60, 7 x 8.4 cm sheets, absorbent filter paper, for use with Mini-PROTEAN or Ready Gel Precast Gels, precut to gel dimensions |
| 1620174 | Immun-Blot® PVDF Membrane, Precut, 7 x 8.4 cm Pkg of 10, 0.2 µm, 7 x 8.4 cm, precut membrane for high binding capacity (150–160 µg/cm) immunoblotting |
| 1450011 | Cell Counting Slides for TC10™/ TC20™ Cell Counter, Dual-Chamber, 30 slides, 60 counts 30 slide pack of dual-chamber slides (60 counts) |
| RDSFAB1849P025 | R&D Systems Human NKp30 Phycoerythrin MAb (Clone 210845), 25 Tests |
| RDSFAB100F025 | R&D Systems Human CD3 epsilon Fluorescein MAb (Clone UCHT1), 25 Tests |
| RDSIC285G025 | R&D Systems Human IFN-gamma Alexa Fluor 488 MAb (Clone 25723), 25 Tests |
| ATCPCS800011 | ATCC Primary Peripheral Blood Mononuclear Cells, Normal, Human BSL1, 1 ml |
| ATCCRL2407 | ATCC NK-92; Natural Killer Cell; Human (Homo sapiens) BSL2, 1 ml |
| ATCTIB152 | ATCC Jurkat, Clone E6-1; Acute T Cell Leukemia; Human (Homo sapiens) BSL1, 1 ml |
| ATC302001 | ATCC RPMI-1640 Medium, 500 ml |
| RDS55001ML | Tocris Protease Inhibitor Cocktail |
| RDS10453IL010 | R&D Systems Recombinant Human IL-2 (Mammalian expressed) Protein CF |
| SEA103579100 | Seahorse XF 200 mM glutamine solution |
| RDSPRD23610 | R&D Systems ProDots Recombinant Human EGF Protein, 50 ug |
| RDS483001K | R&D Systems TACS Annexin V-FITC Apoptosis Detection Kit |
| RDSFMC021 | R&D Systems FlowX Human Regulatory T Cell MultiColor Flow Kit |
| RDSAF231SP | R&D Systems Human EGF R/ErbB1 Affinity Purified Polyclonal Ab |
| RDSMAB1799SP | R&D Systems Human/Mouse/Rat STAT3 MAb (Clone 232209) |
| RDSAF5718SP | R&D Systems Human/Mouse/Rat GAPDH Affinity Purified Polyclonal Ab |
| RDSHAF008 | R&D Systems Goat Anti-Rabbit IgG HRP Affinity Purified PAb |
| RDSHAF007 | R&D Systems Goat Anti-Mouse IgG HRP Affinity Purified PAb |
| RDSHAF109 | R&D Systems Donkey Anti-Goat IgG HRP Affinity Purified PAb |
| NOVNXA6050 | Novus RunBlue Prestained Molecular-Weight Marker Tri colour ,500ul |
| NOVNBP29881450UL | Novus PIK3CA Antibody |
| POL101000028 | Polyplus INTERFERin® |
| FAL353090 | Falcon Cell Culture insert for 6 well Plate with 0.4 µm Pore Transparent PET membrane, 1/Pack, 48/Case |
| FAL353091 | Falcon Cell Culture insert for 6 well Plate with 3.0 µm Pore Transparent PET membrane, 1/Pack, 48/Case |
| 4906845001 | PhosSTOP™ |
| F0895-2MG | Fibronectin human plasma |
| T8154-20ML | Trypan Blue solution |
| HPA026652-25UL | Anti-CCDC88B antibody produced in rabbit |
| D5671-500ML | Dulbecco’s Modified Eagle’s Medium - high glucose |
| 91077C-100MG | Insulin, Human Recombinant |
| T4049-100ML | Trypsin-EDTA solution |
| A6964-500ML | Accutase® solution |
| P7170-1L | Ponceau S solution |
| 5150 | MyeloCult H5100 |
| 74142 | Hydrocortisone |
| 7930 | Cryostor CS10 |
| AM16704 | PRE-DESIGNED SIRNA, STD 20NMOL EACH |
| A1286301 | PBS (FLOW CYTOMETRY GRADE) 10L |
| A3161001 | FBS QUALIFIED AUSTRALIA ORIGIN 50 ML |
| 10006D | IMMUNOPRECIPITATION KIT - DYNA 2 ML KIT |
| A1443001 | DMEM WO GLUC & PHENOL RED 500mL |
| T8326 | UM-HACC-2A Cells |
| RDSARY009 | R&D Systems Proteome Profiler Human Apoptosis Array Kit |
| RDSARY003C | R&D Systems Proteome Profiler Human Phospho-Kinase Array Kit |


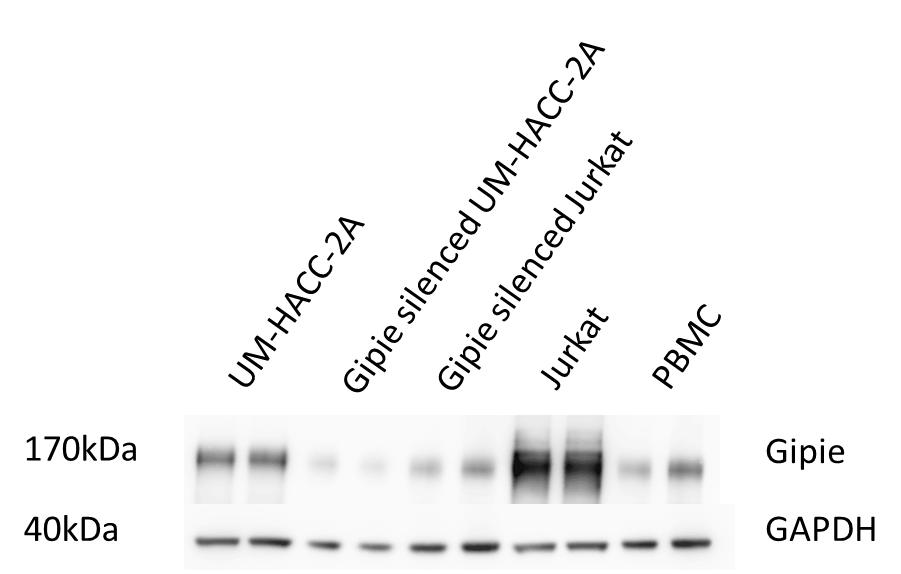


**Supplementary Figure 1. Western Blot image of UM-HACC-2A, Jurkat, and PBMC showing expression and silencing of Gipie.** The loading amount was 20µg. Gipie western blot staining was performed using Anti-CCDC88B antibody (1:400) and GAPDH Affinity Purified Polyclonal Ab (1 µg/ml).


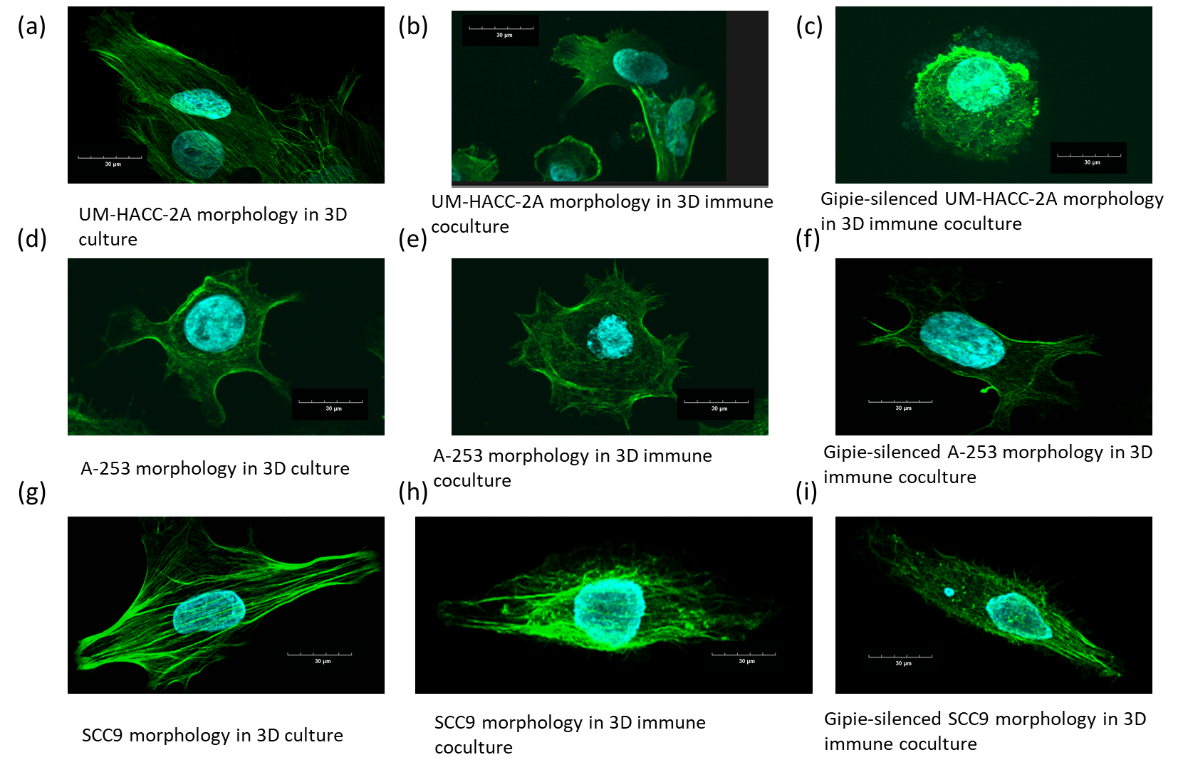


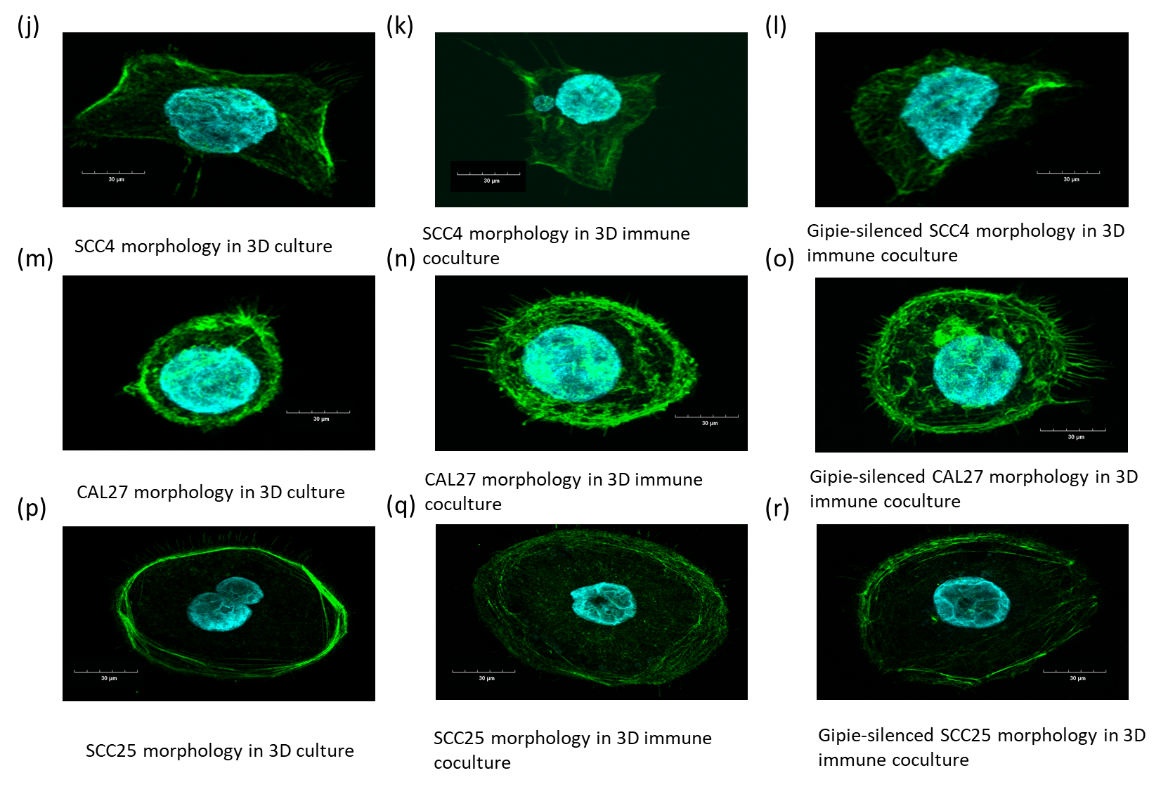


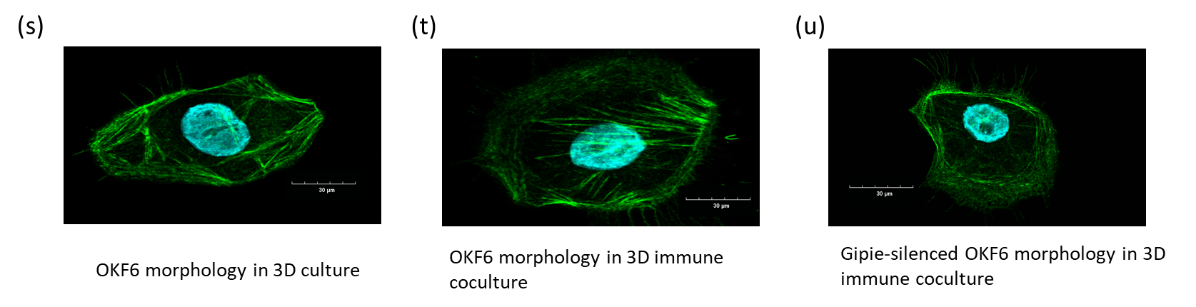


**Supplementary Figure 2. Images of all cell lines in different conditions.** Actin (green) and DAPI (blue) counterstained 3D z stack confocal images at 100 X magnification, n = 24 biological replicates at different passage numbers. Scale bar 30 µm.

**Supplementary Figure 3. Graphical representation of annexin V flow cytometry in NK models.** Graph shows percentage apoptotic cells multiple comparison of UM-HACC-2A, A-253, SCC4, SCC9, SCC25, CAL 27, OKF6, and their respective Gipie-silenced counterparts, all in JK coculture model. One-way ANOVA Kruskal-Wallis multiple comparison graph made with Graph Pad Prism 9.4.1. *p ≤ 0.05, **p ≤ 0.005, ***p ≤ 0.0001, ****p < 0.0001. n = 24 biological replicates at different passage numbers.

**Supplementary Figure 4. Graphical representation of annexin V flow cytometry in JK models.** Graph shows percentage apoptotic cells multiple comparison of UM-HACC-2A, A-253, SCC4, SCC9, SCC25, CAL 27, OKF6, and their respective Gipie-silenced counterparts, all in JK coculture model. One-way ANOVA Kruskal-Wallis multiple comparison graph made with Graph Pad Prism 9.4.1. *p ≤ 0.05, **p ≤ 0.005, ***p ≤ 0.0001, ****p < 0.0001. n = 24 biological replicates at different passage numbers.


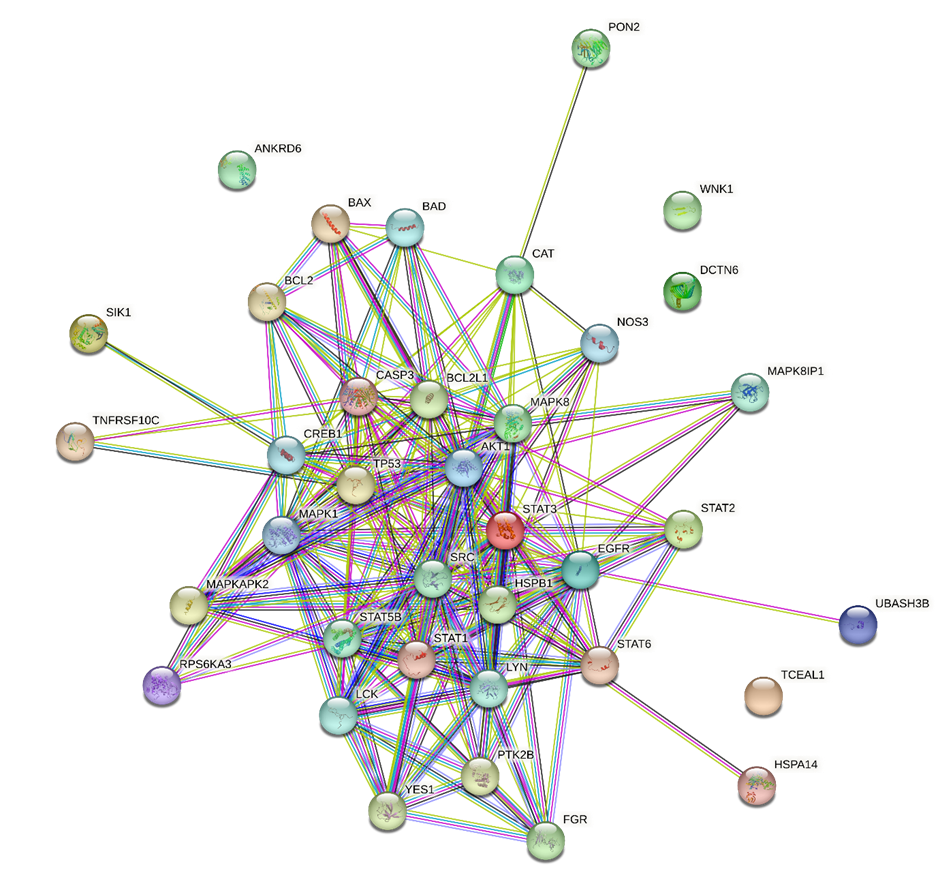


**Supplementary Figure 5. Protein interaction network representation.** STRING protein-protein interaction network generated using STRING functional protein association networks <https://string-db.org/>. The interaction network of 33 phosphorylated proliferation mechanism and apoptotic pathways related proteins significantly affected by Gipie in adenoid cystic carcinoma. Number of nodes 37, number of edges 212, average node degree 11.5, and average local clustering coefficient 0.753.


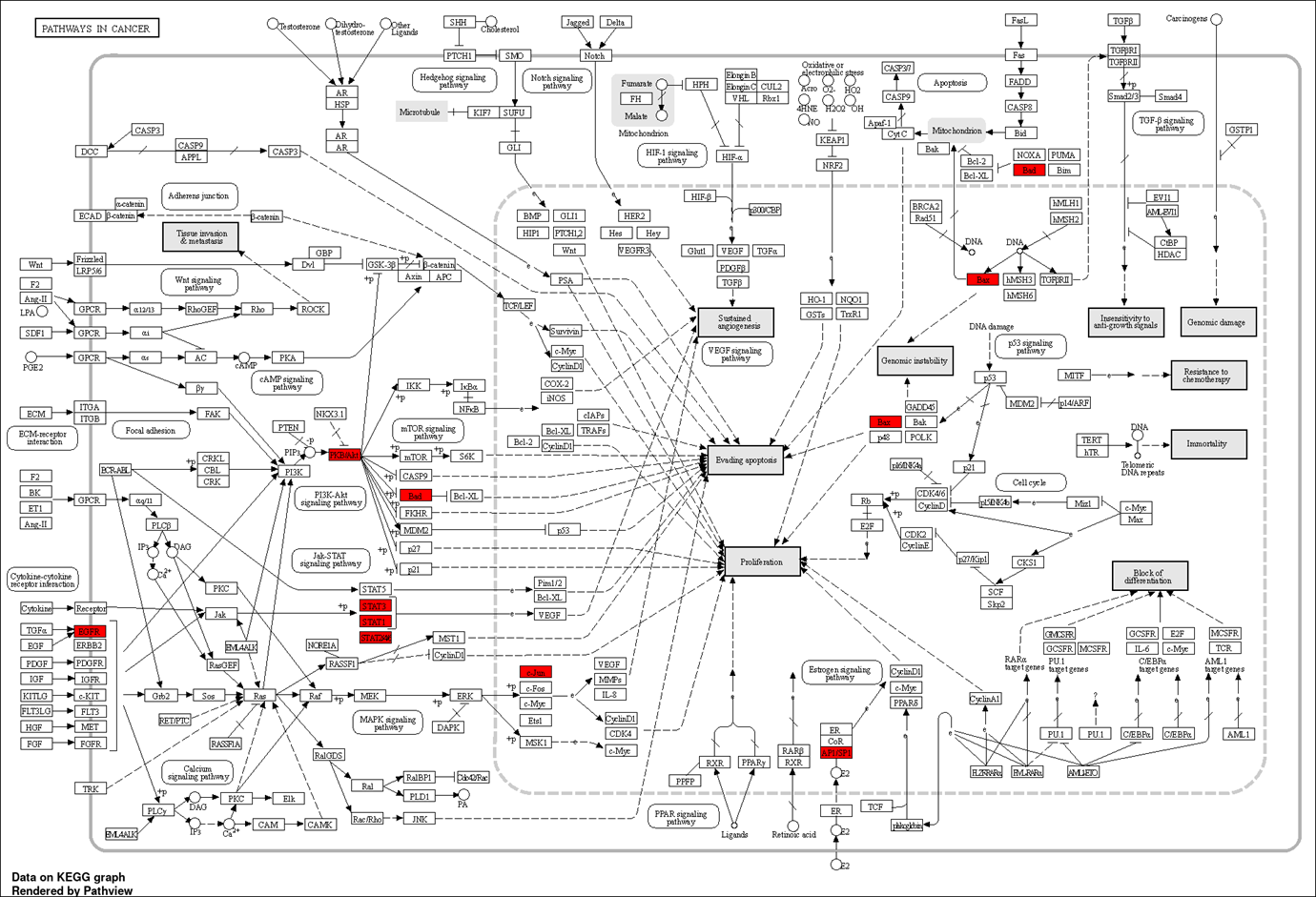


**Supplementary Figure 6. Proliferation pathways of Cancer.** KEGG generated schematic representation of proliferation mechanism impacted by Gipie. The generated pathway is based on phosphoproteomic analysis of phosphorylated proliferation mechanism protein affected by Gipie in UM-HACC-2A. The affected proteins are EGFR, AKT, STAT3, STAT1, STAT4, cJUN, BAD, and BAX. The KEGG pathway was made using the database for annotation, visualization, and integrated discovery (DAVID) <https://david.ncifcrf.gov/>.


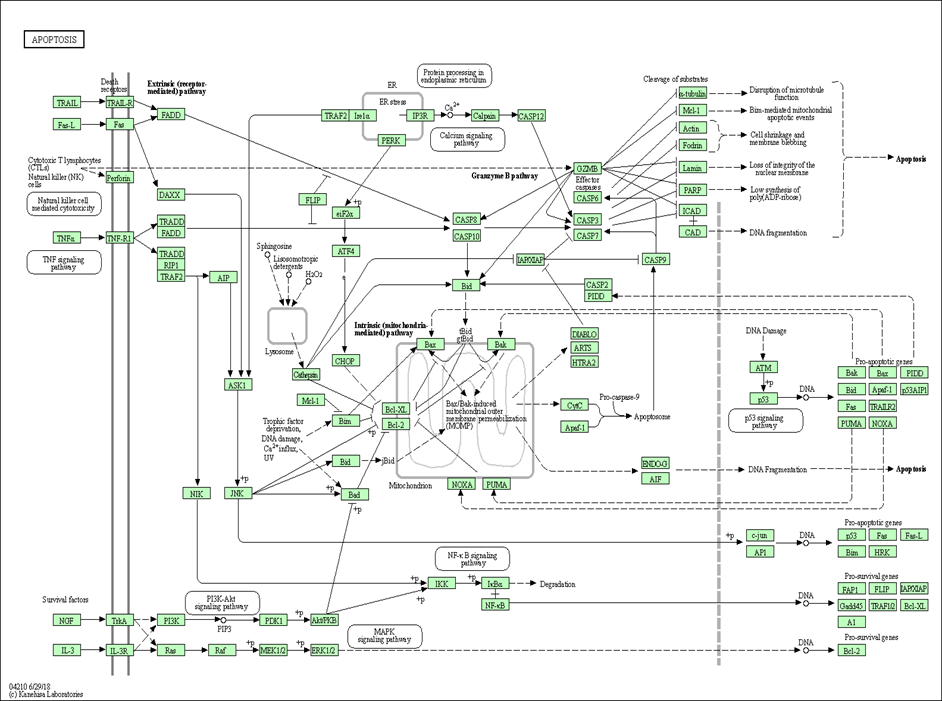


**Supplementary Figure 7. Apoptosis pathways of Cancer.** KEGG generated schematic representation of apoptosis affected proteome impacted by Gipie. The generated pathway is based on phosphoproteomic analysis of phosphorylated proliferation mechanism protein affected by Gipie in UM-HACC-2A. The affected proteins are Fas-L, TRAIL, TNFα, NOF, and IL3. The KEGG pathway was made using the database for annotation, visualization, and integrated discovery (DAVID) <https://david.ncifcrf.gov/>.


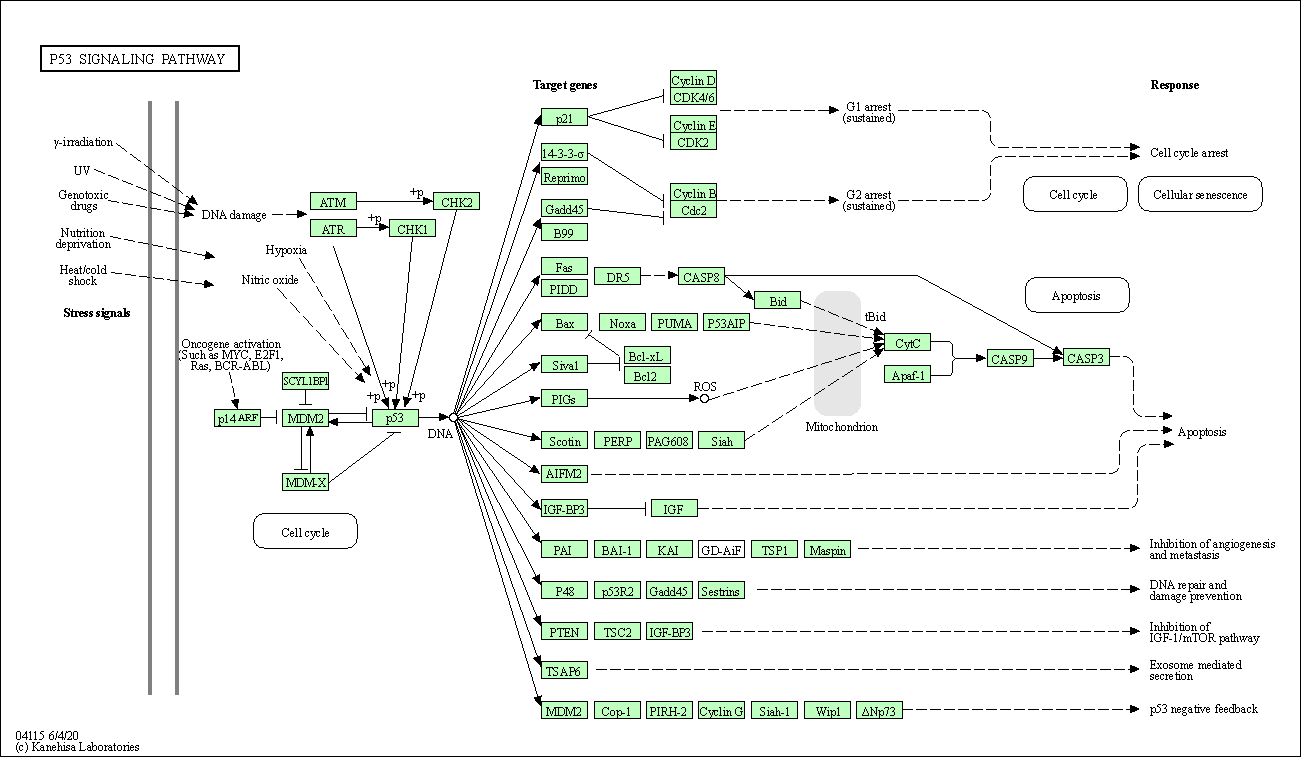


**Supplementary Figure 8.** **p53 mediated apoptosis pathways of Cancer.** KEGG generated schematic representation of proliferation mechanism impacted by Gipie. The generated pathway is based on phosphoproteomic analysis of phosphorylated proliferation mechanism protein affected by Gipie in UM-HACC-2A. The KEGG pathway was made using the database for annotation, visualization, and integrated discovery (DAVID) <https://david.ncifcrf.gov/>.


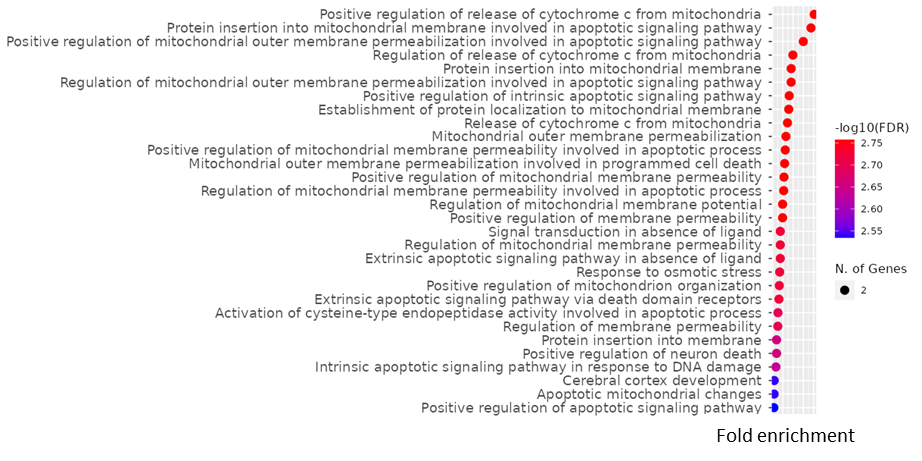


**Supplementary Figure 9. Dot plot analysis of overrepresented Gipie-affected anti-apoptotic and proapoptotic proteins related pathways in UM-HACC-2A.** The size of the dot represents the fold enrichment, while its colour represents the FDR (p adjusted) value for each enriched Reactome pathway (high: red, low: blue). The x-axis represents the percentage of upregulated proteins in the selected pathway that is presented in the y-axis.


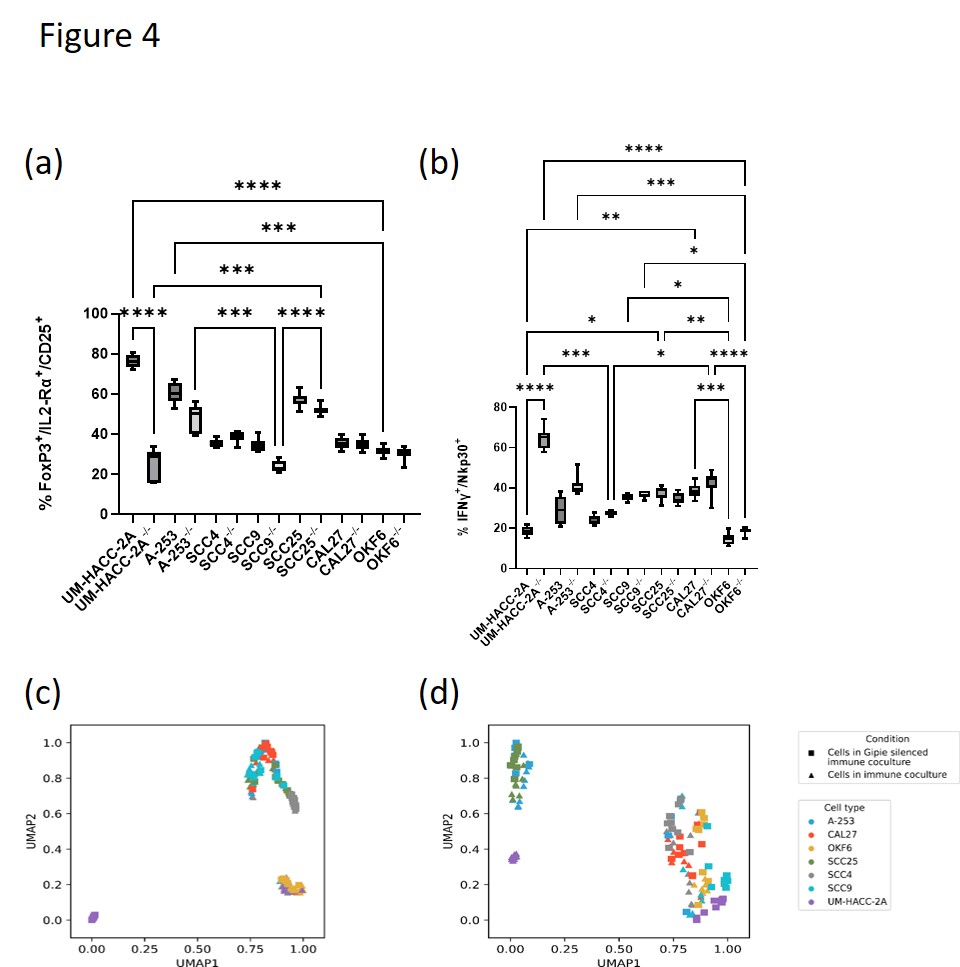


**Supplementary Figure 10. Gipie affected antitumour activity of immune cells against adenoid cystic carcinoma.** (a) & (b) Graph shows T regulatory cells flow cytometry-based percentage FoxP3^+^/IL-2Rα^+^/CD25^+^ cells and NK activated cells flow cytometry-based percentage IFN-γ^+^/ NKp30^+^ cells multiple comparison of UM-HACC-2A, A-253, SCC4, SCC9, SCC25, CAL27, OKF6, and their respective Gipie-silenced counterparts, all in JK and NK co-culture model respectively. One-way ANOVA Kruskal-Wallis multiple comparison graph made with Graph Pad Prism 9.4.1. *p ≤ 0.05, **p ≤ 0.005, ***p ≤ 0.0001, ****p < 0.0001. n = 24 biological replicates at different passage numbers, (c) & (d) Two-dimensional representations learnt by UMAP on the percentage of late apoptotic cells in JK and NK cell co-cultures, and either (c) the percentage of cells expressing FoxP3^+^ cells and FoxP3^+^/IL2-Rα^+^/CD25^+^ cells, or (d) the percentage of cells expressing IFNγ^+^ cells and IFNγ^+^/Nkp30^+^ cells. Each point represents either cells in Gipie-silenced immune co-culture (square), or cells in immune co-culture (triangle), and points are highlighted based on their cell type. We used the UMAP implementation of the umap-learn Python module.


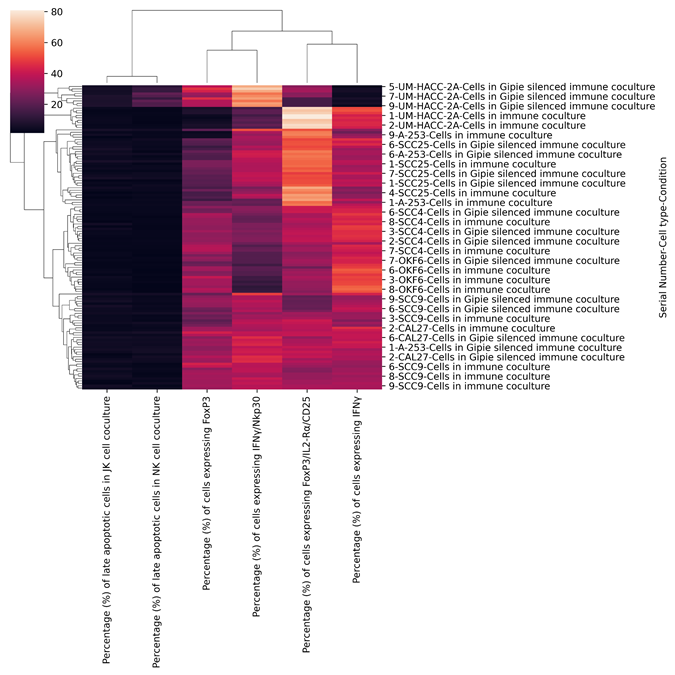


**Supplementary Figure 11. Visualization of all marker expressions in all cell lines in different treatment conditions.** Heat map to visualize hierarchical clustering of percentage of late apoptotic cells in JK and NK cell coculture, percentage of cells expressing FoxP3, FoxP3/IL-2Rα/CD25, IFN-γ/ NKp30, and IFN-γ in all the oral and salivary gland cancer cells (unaltered and Gipie-silenced) is made with R (version 4.1.1) in RStudio (R version 4.1.1, 2021-08-10), heatmap.2() [gplots R package 3.1.3] and visualized using the ‘ggplot2’ package. The protein intensities are log10 transformed and are displayed as colours ranging from deep purple to orange as shown in the key. Both rows and columns are clustered using correlation Euclidean distance measures and average linkage.


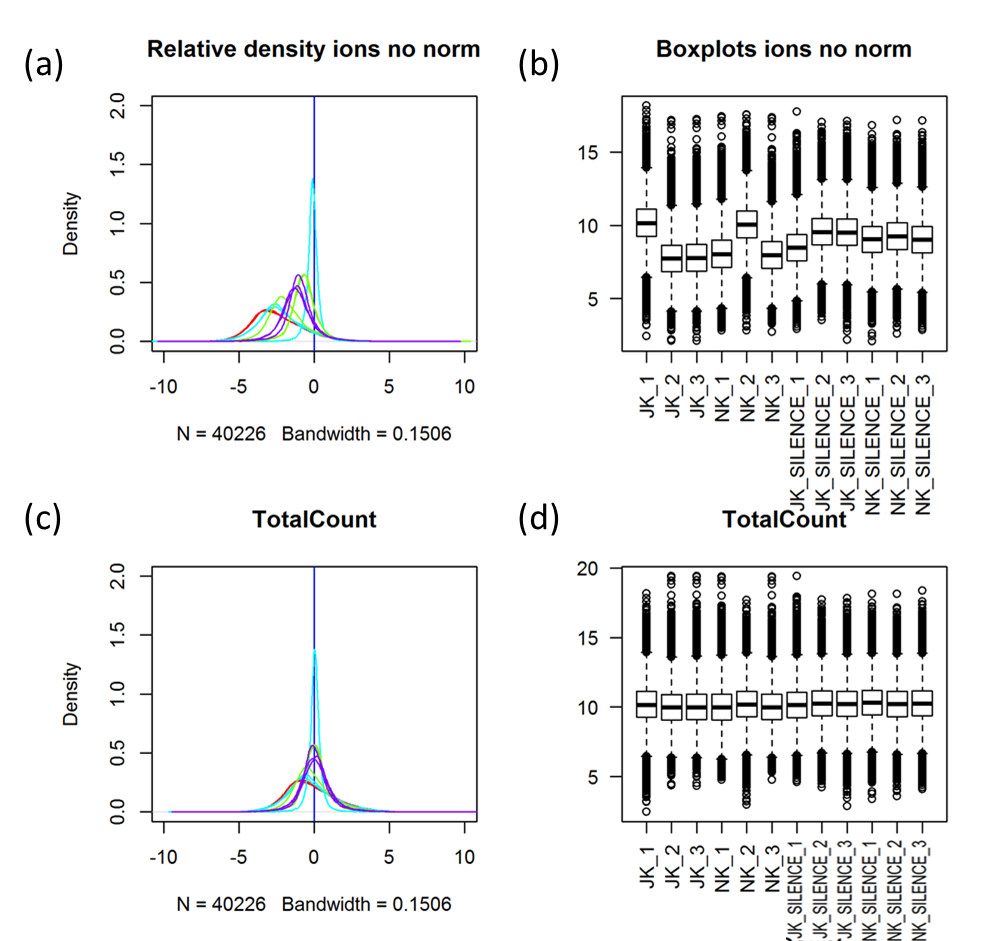


**Supplementary Figure 12. SWATH of immune cells in co-culture model overall data.** Contains density plots of the relative ion ratios with respect to the first sample, and boxplots of log ion peak areas, log transformed or normalized. (a) relative ion density plot, (b) log transformed of log ion peak area of immune cells. It indicates that the protein expressions (based on ion peak area) in the immune cells from Gipie-silenced UM-HACC-2A coculture models were higher compared to protein expressions in the immune cells from its normal counterpart, (c) normalised density plot of relative ion ratio, and (d) normalised total count box plot.


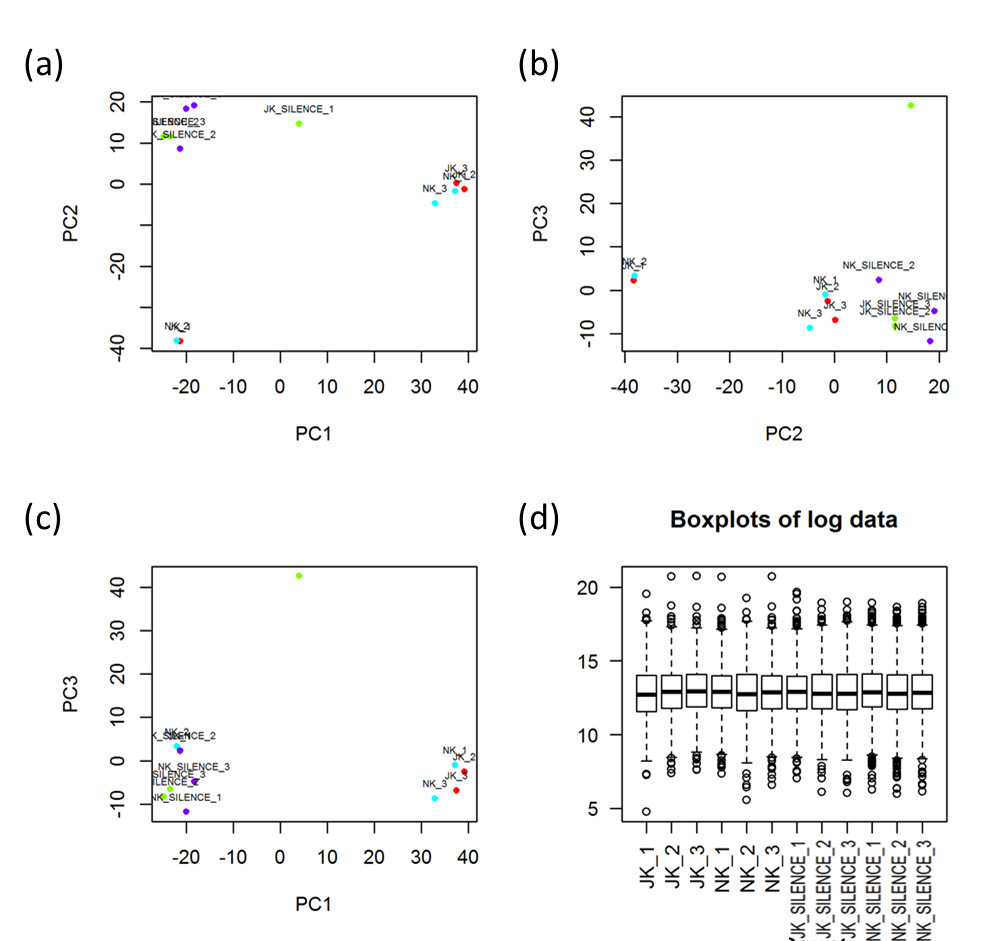


**Supplementary Figure 13. PCA 2D plots of immune activation LC-MS/MS.** Plots from a principal component analysis carried out on normalized protein data, and a boxplot of the normalized protein level data. (a) PC2 versus PC1, (b) PC3 versus PC2, (c) PC3 versus PC1, (d) box plot showing normalised protein level data pf all immune cells and respective treatment conditions. The box plot exhibits same amount of protein analysed for all the groups. Therefore, no presence of data analysis discrepancies.


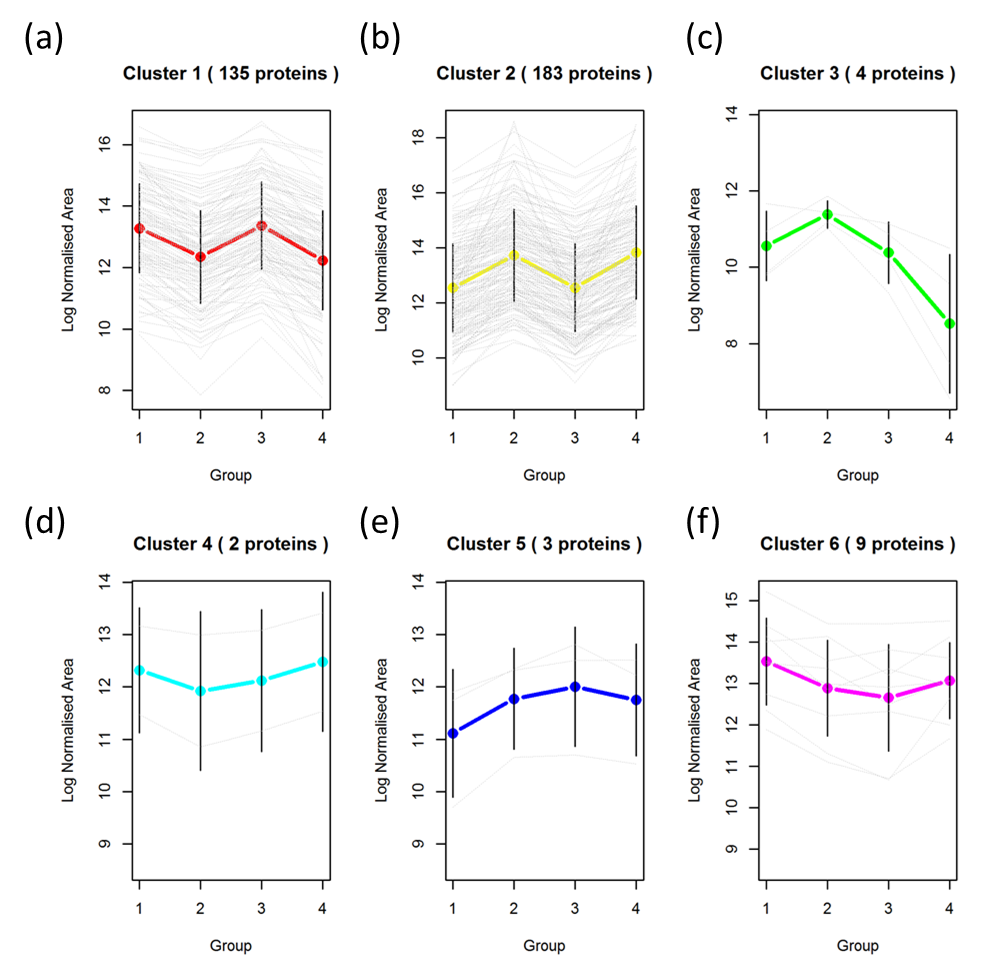


**Supplementary Figure 14. Protein expression cluster profiles.** Shows the expression patterns of individual proteins, likewise only using those differentially expressed based on the ANOVA analysis. GenePattern server (*SwathPairsAndOverall*) was used to identify proteins with significant change of protein expressions in four experimental groups, JK, JK cells from Gipie-silenced UM-HACC-2A coculture, NK, and NK cells from Gipie-silenced UM-HACC-2A coculture. The protein expression profiles were grouped into six clusters that showed distinct expression profiles, and the median expression of all proteins in the cluster was plotted for each experimental condition.

**
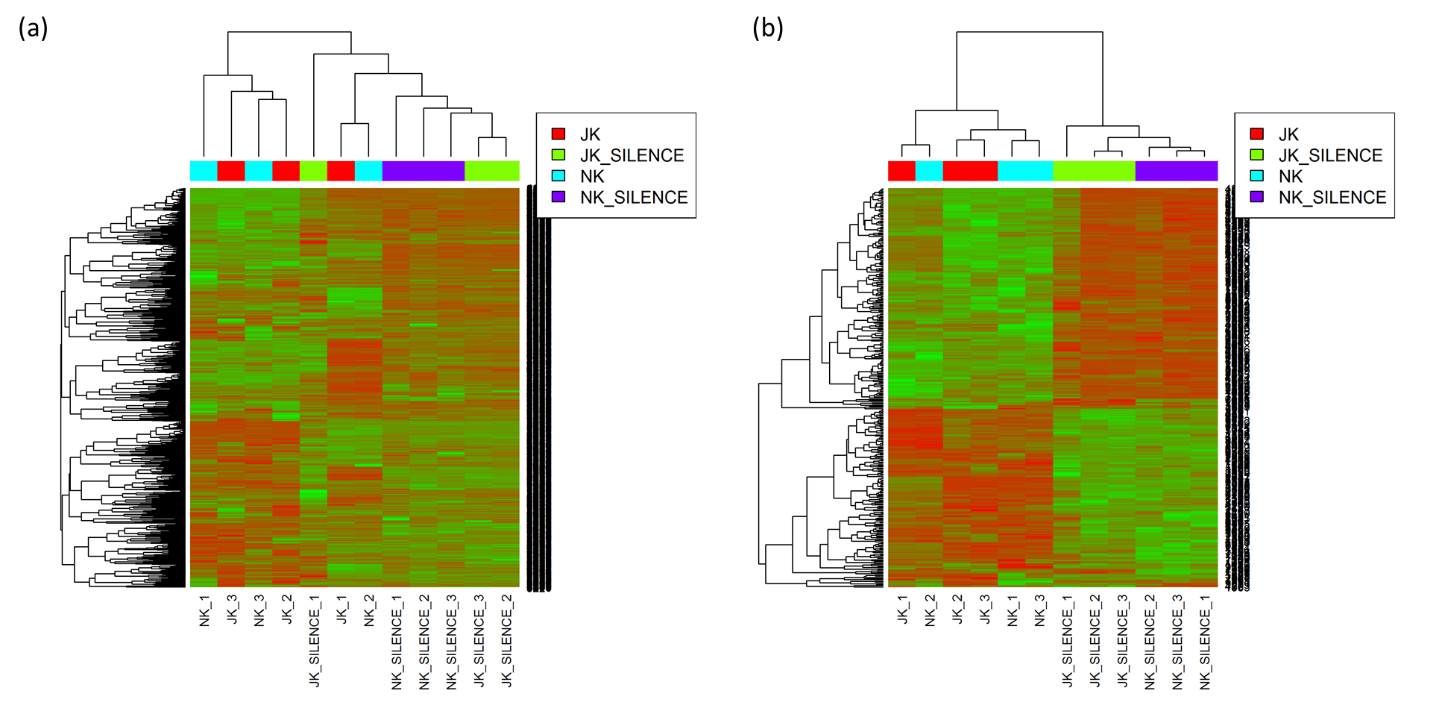
**

**
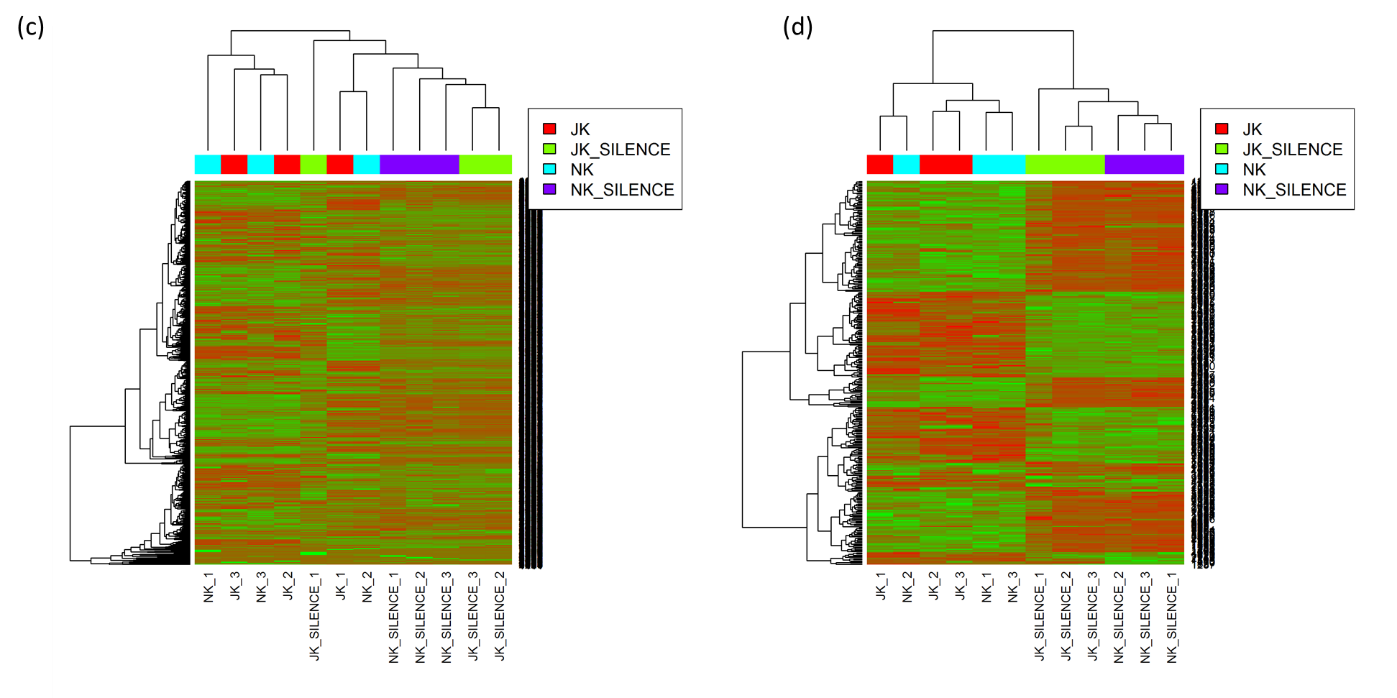
**

**Supplementary Figure 15. DIA SWATH analysis of immune cells from UM-HACC-2A coculture models** (a) Heatmap cordist all; correlation-based metric, (b) Heatmap cordist - ANOVA DE, (c) Heatmap Euclidean all contains the heatmap of all proteins (Euclidean metric), (d) heatmap Euclidean ANOVA DE. ANOVA DE contains the heatmap of the differentially expressed proteins from the ANOVA analysis (p-value < cut off 0.05 and Max FC > cut off 1.5) protein expression fold change Group 1/Group 2 for each pairwise comparison.


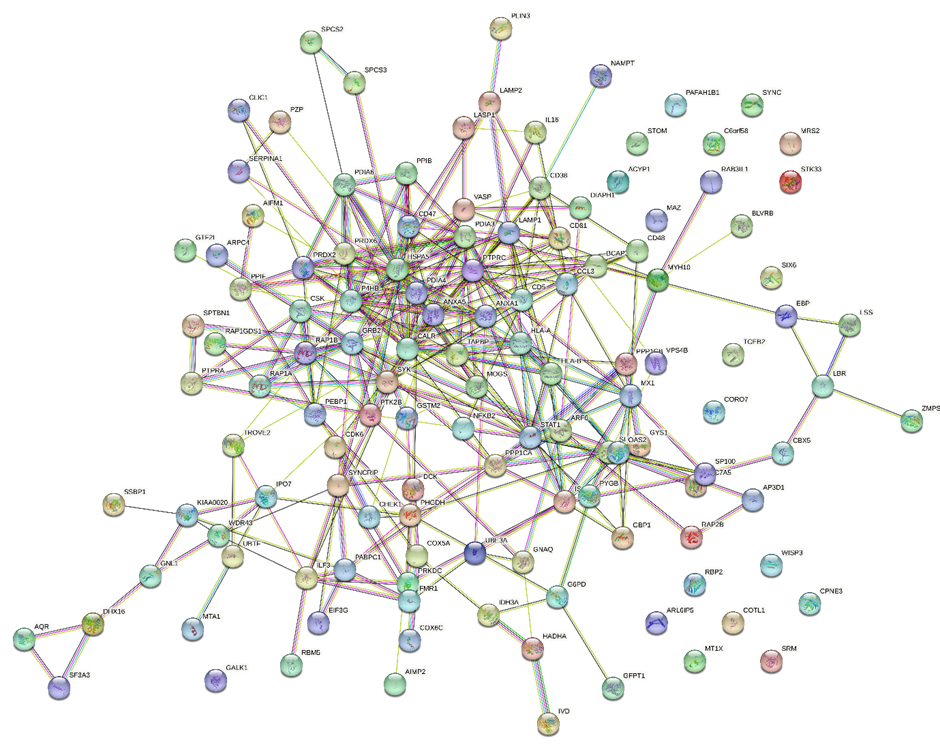


**Supplementary Figure 16. Protein interaction network representation of Gipie-affected proteins in immune cells.** STRING protein-protein interaction network generated using STRING functional protein association networks <https://string-db.org/>. All components are not joined based on ranks. Thus, the network was transferred to Cytoscape 3.9.1 [www.cytoscape.org](http://www.cytoscape.org).


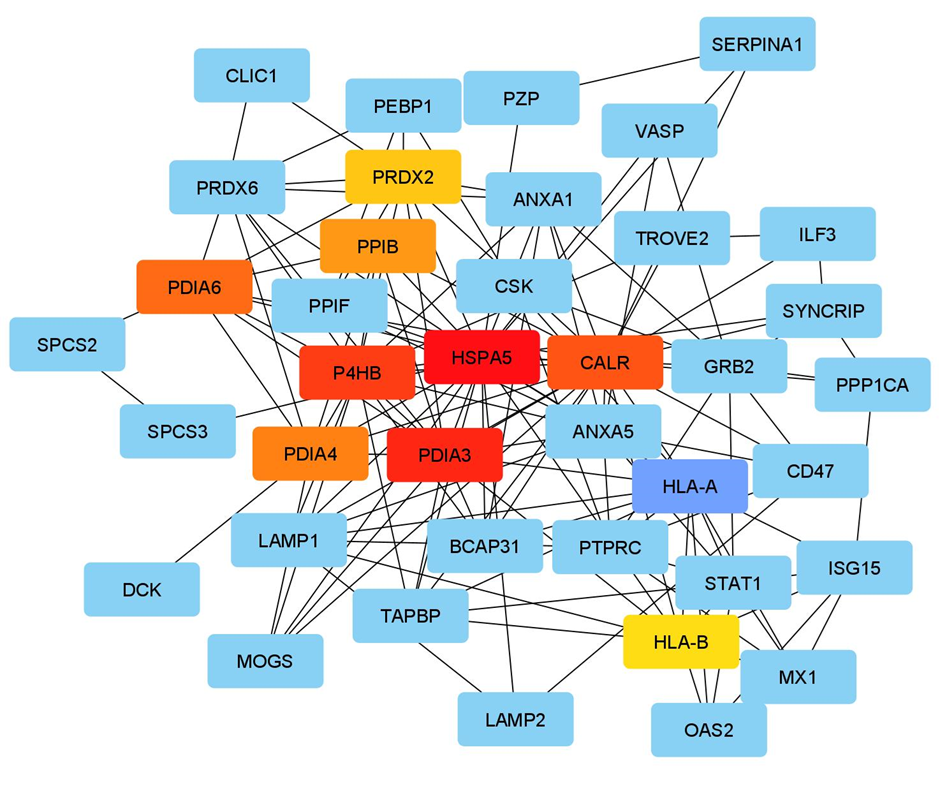


**Supplementary Figure 17**. **SWATH LC-MS/MS analysis.** Top 200 significant altered protein in immune activated cells transferred to STRING <https://string-db.org/>; Tab separated values filtered based on score ≥0.4; exported to Cytoscape; model construction and style modification completed after top 75 nodes based on Matthews correlation coefficient (MCC) and displayed first-stage nodes, expanded subnetwork, and the shortest path. The degree of closeness of each node (represents each protein) is displayed as colours ranging from blue (low) to red (high).


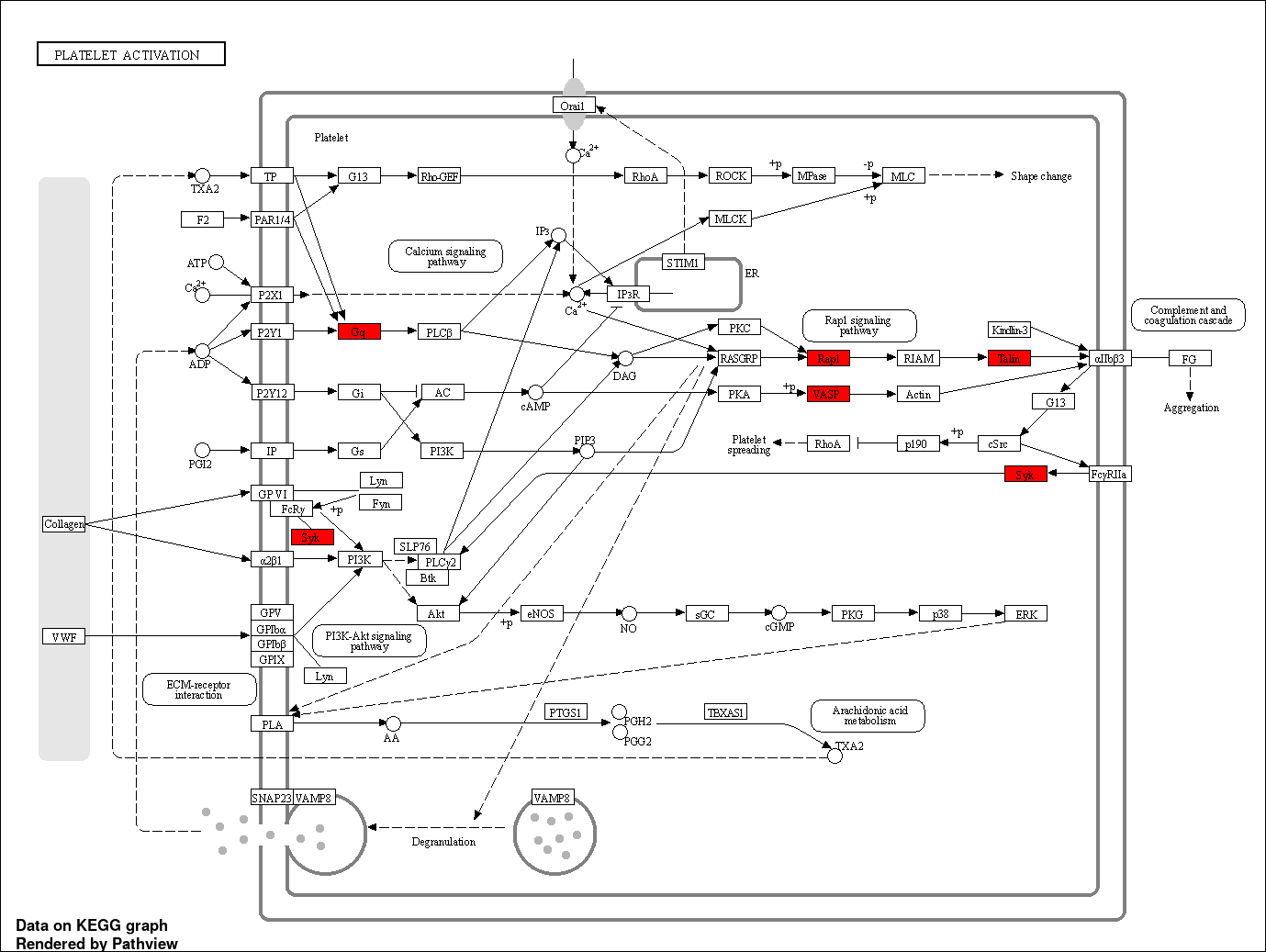


**Supplementary Figure 18. Platelet activation pathway.** KEGG generated schematic representation of platelet activation pathway impacted by Gipie in immune cells. The generated pathway is based on DIA SWATH analysis of differentially expressed proteins in immune cells due to silencing of Gipie in UM-HACC-2A coculture models. The Gipie-affected immune cell proteins are marked in red. The KEGG pathway was made using the database for annotation, visualization, and integrated discovery (DAVID) <https://david.ncifcrf.gov/>.


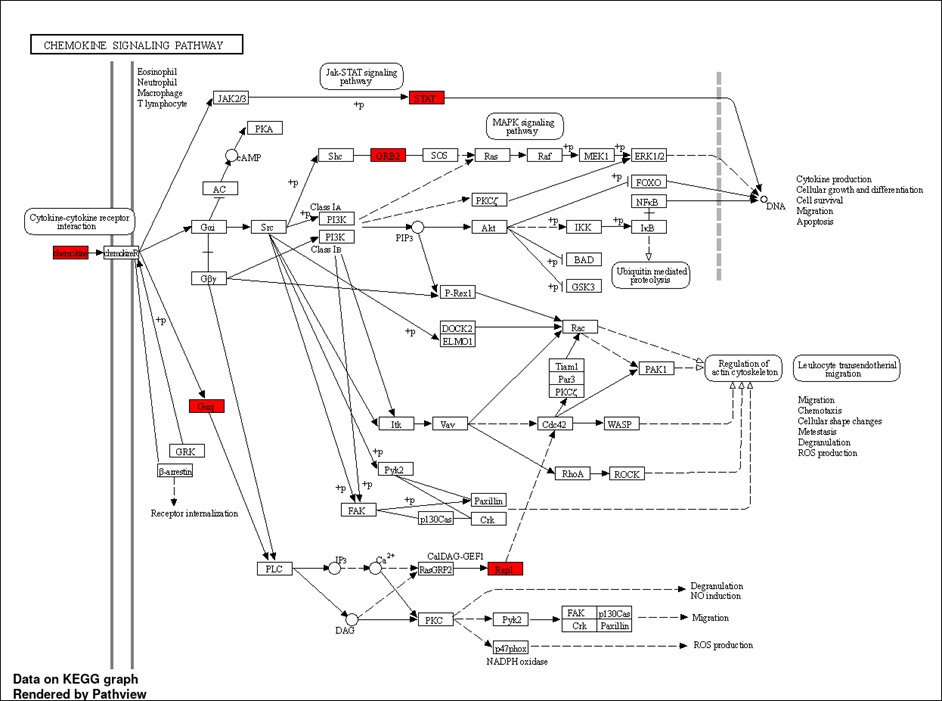


**Supplementary Figure 19. Chemokine signalling pathway.** KEGG generated schematic representation of chemokine signalling pathway impacted by Gipie in immune cells. The generated pathway is based on DIA SWATH analysis of differentially expressed proteins in immune cells due to silencing of Gipie in UM-HACC-2A coculture models. The Gipie-affected immune cell proteins are marked in red. The KEGG pathway was made using the database for annotation, visualization, and integrated discovery (DAVID) <https://david.ncifcrf.gov/>.


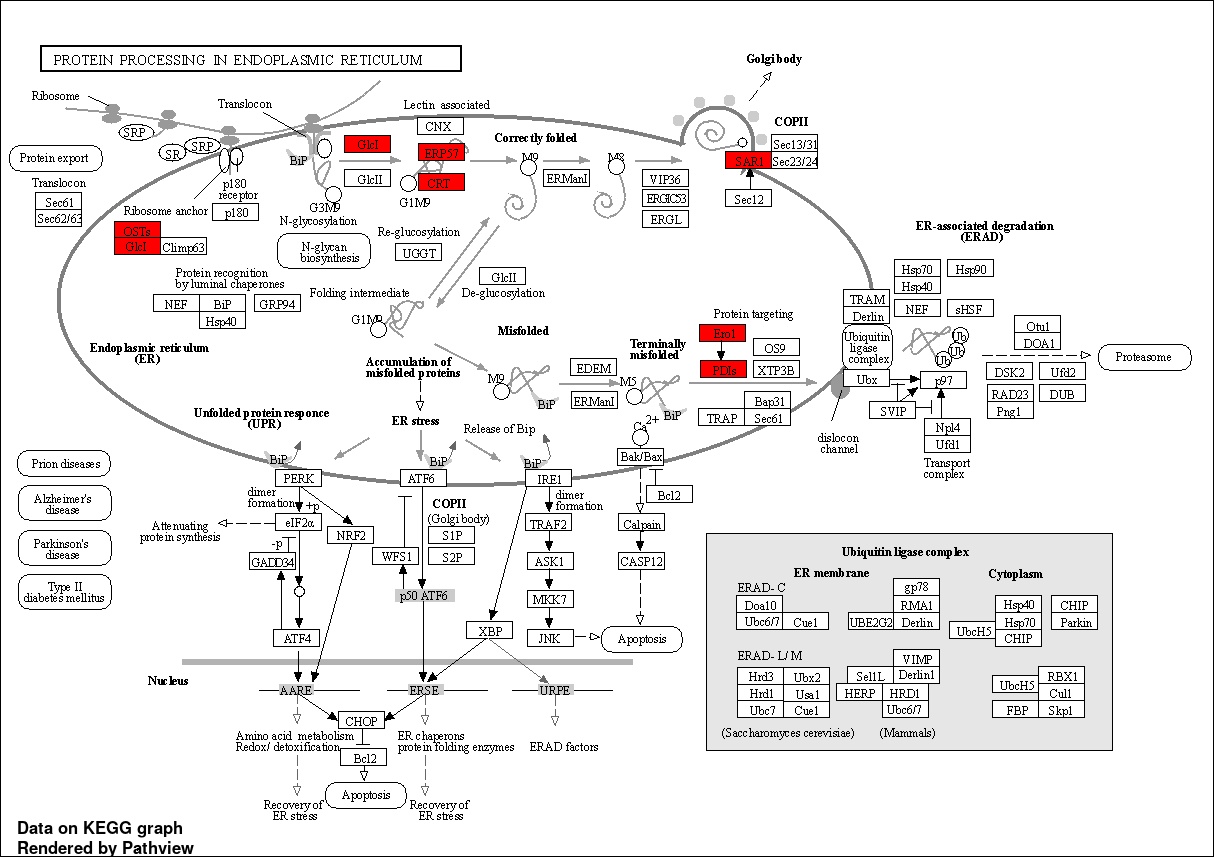


**Supplementary Figure 20. Protein processing in endoplasmic reticulum.** KEGG generated schematic representation of protein processing in endoplasmic reticulum impacted by Gipie in immune cells. The generated pathway is based on DIA SWATH analysis of differentially expressed proteins in immune cells due to silencing of Gipie in UM-HACC-2A coculture models. The Gipie-affected immune cell proteins are marked in red. The KEGG pathway was made using the database for annotation, visualization, and integrated discovery (DAVID) <https://david.ncifcrf.gov/>.
